# Thermal stability of single‐domain antibodies estimated by molecular dynamics simulations

**DOI:** 10.1002/pro.3546

**Published:** 2018-12-20

**Authors:** Gert‐Jan Bekker, Benson Ma, Narutoshi Kamiya

**Affiliations:** ^1^ Institute for Protein Research, Osaka University Suita Osaka Japan; ^2^ School of Computational Science and Engineering Georgia Institute of Technology Atlanta Georgia 30332; ^3^ Graduate School of Simulation Studies University of Hyogo Kobe Hyogo Japan

**Keywords:** molecular dynamics simulation, single‐domain antibody, thermal stability, melting temperature, point mutations

## Abstract

Single‐domain antibodies (sdAbs) function like regular antibodies, however, consist of only one domain. Because of their low molecular weight, sdAbs have advantages with respect to production and delivery to their targets and for applications such as antibody drugs and biosensors. Thus, sdAbs with high thermal stability are required. In this work, we chose seven sdAbs, which have a wide range of melting temperature (*T*_m_) values and known structures. We applied molecular dynamics (MD) simulations to estimate their relative stability and compared them with the experimental data. High‐temperature MD simulations at 400 K and 500 K were executed with simulations at 300 K as a control. The fraction of native atomic contacts, *Q*, measured for the 400 K simulations showed a fairly good correlation with the *T*_m_ values. Interestingly, when the residues were classified by their hydrophobicity and size, the *Q* values of hydrophilic residues exhibited an even better correlation, suggesting that stabilization is correlated with favorable interactions of hydrophilic residues. Measuring the *Q* value on a per‐residue level enabled us to identify residues that contribute significantly to the instability and thus demonstrating how our analysis can be used in a mutant case study.

## Introduction

The variable fragment of antibodies (*F*_v_), which consists of heavy (*V*_H_) and light chains (*V*_L_), binds to antigens with high specificity and affinity. Each chain has three complementarity‐determining region (CDR) loops (i.e., CDR‐H1, CDR‐H2, and CDR‐H3 in *V*_H_ and CDR‐L1, CDR‐L2, and CDR‐L3 in *V*_L_), which play an important role in antigen binding. Among them, the CDR‐H3 loop, which is diverse in both sequence length and composition, is the most crucial component for recognizing antigens. The other part of *F*_v_ is called the framework, which contributes to stabilize their structures by inter‐ and intra‐chain interactions. The framework forms an immunoglobulin fold with a β‐sandwich motif, where two β‐sheet structures face each other. Since *F*_v_ consists of two chains, they unfold and aggregate at high temperature (> 60°C).^1^

A different type of antibody is the heavy chain antibody (hcAb), where the variable domain only consists of the heavy chain (*V*_H_H). These hcAbs have been identified in llama, camelids and sharks, from which the *V*_H_H were cloned and expressed.^2^, ^3^ By only cloning the *V*_H_H, single‐domain antibodies (sdAbs) were obtained, which were shown to be more stable than conventional antibodies and have the ability to reversibly refold to their native structure after being denatured by heating.^4^ Similar to the case of V_H_H, sdAbs can also be obtained from the *V*_H_ and *V*_L_ of regular antibodies. Due to their smaller size, sdAbs have advantages with respect to production, solubility, and delivery to their targets and are shaping up to become promising experimental and therapeutic tools.^5^ For practical applications of sdAbs such as antibody drugs and biosensors, it is important to understand factors that affect their thermal stability.

**Figure 1:**
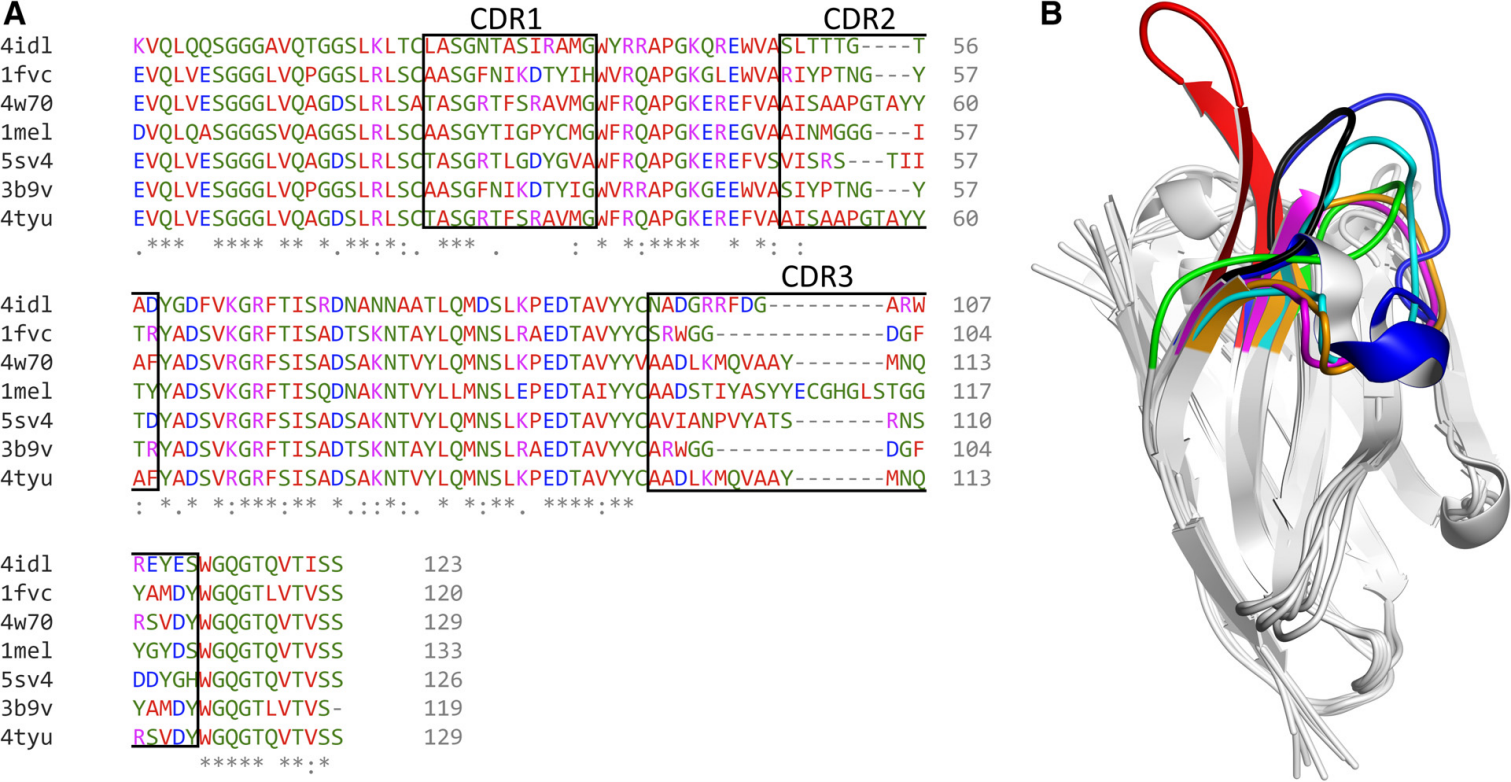
(A) Multiple sequence alignment of 4idl, 1fvc, 4w70, 1mel, 5sv4, 3b9v, and 4tyu performed by Clustal Omega,^13^ where the consensus symbols shown below the alignment and the residue colors are the defaults used by Clustal Omega. The location of the CDR loops is indicated in the figure. (B) Superposition of 4idl, 1fvc, 4w70, 1mel, 5sv4, 3b9v, and 4tyu with the CDR3 loop in red, black, orange, blue, cyan, green, and magenta, respectively. The image was drawn by Molmil,^14^ a WebGL molecular viewer developed by Protein Data Bank Japan.^15^, ^16^

Although a considerable number of crystal structures of sdAbs have been registered in the Protein Data Bank (PDB),^6^ very few structures have had their melting temperatures (*T*_m_) measured. Recently, crystal structures of sdAbs from llama and camelid, which exhibit an unexpectedly low *T*_m_ (47°C, [PDB ID 4idl^7^]) and very high *T*_m_ (85°C, PDB ID 4tyu^8^), respectively, have been determined (Table 1). Furthermore, antibodies consisting of only the *V*_H_ have been engineered from conventional antibodies by mutating the amino acid residues in the interface to the *V*_L_. Here, Barthelemy et al. took an existing antibody [PDB ID 1fvc^9^] and was able to engineer it into an sdAb [PDB ID 3b9v^12^] with improved stability, raising the *T*_m_ from 58°C to 79°C (Table 1). Figure 1 shows several superposed *V*_H_ domains of antibodies and sdAbs, where the structure in the framework region forming the β‐sandwich motif, are similar, while the CDR loops are diverse.

**Table 1:** The Dataset of sdAbs Used in this Work

PDB ID	Resolution (Å)	*T*_m_ (°C)	CDR1 range	CDR2 range	CDR3 range
4idl^7^	2.09	46.75	Leu23–Gly35	Ser50–Asp58	Asn96–Ser112
1fvc^9^	2.20	58.00	Ala23–His35	Arg50–Arg59	Ser97–Tyr109
4w70^8^	2.28	60.00	Thr23–Gly35	Ala50–Phe62	Ala100–Tyr118
1mel^10^	2.50	69.00	Ala23–Gly35	Ala50–Tyr59	Ala97–Ser122
5sv4^11^	2.70	70.70	Thr23–Ala35	Val50–Asp59	Ala97–His115
3b9v^12^	1.80	79.00	Ala23–Gly35	Ser50–Arg59	Ala97–Tyr109
4tyu^8^	2.13	85.00	Thr23–Gly35	Ala50–Phe62	Ala100–Tyr118

*Note*: List of protein structures used in this work obtained from Protein Data Bank Japan with their respective resolution, *T*_m_ value and the CDR loop ranges standardized to the numbering used in this work. The original sequence numbering of 3b9v in CDR2 and CDR3 correspond to Ser50–Ile51–Tyr52–Pro52A–Thr53–Asn54–Gly55–Tyr56–Thr57–Arg58 and Ala93–Arg94–Trp95–Gly96–Gly97–Asp98–Gly99–Phe100–Tyr100A–Ala100B–Met100C–Asp101–Tyr102, respectively.

Since molecular dynamics (MD) simulations can reproduce protein motions and protein–target interactions at atomic resolution, they have been widely applied to antibody systems, especially for rational antibody design (see the review by Yamashita^17^). We previously predicted the structure of the CDR‐H3 loop in a conventional antibody by using MD simulations^18^ and used MD for the refinement and the ranking of decoys generated by rigid‐body antibody–antigen docking.^19^ Olson et al. measured the thermal stability of sdAbs using an enhanced MD method with an implicit solvent model to compare experimental and modeled structures, and was able to show qualitative agreement between the two.^20^ Although the *T*_m_ is defined as the temperature at which folded and unfolded proteins are in equilibrium, it can be difficult to measure both folded and unfolded states at atomic resolution using experimental methods. While MD simulations can measure temperature dependence of structures directly, the required simulation time to measure this is not tractable for a high‐throughput protocol.

Consequently, it is not practical to measure the *T*_m_ by MD simulations at or near the experimental *T*_m_ (e.g., 50°C). On the other hand, high‐temperature MD simulations (e.g., at 400 K) could provide us valuable information for protein design, such as which residues are flexible and whether native‐like interactions are preserved or not.^21^ We previously employed such high temperature simulations to qualitatively estimate the stability of predicted binding configurations between an enzyme, cyclin‐dependent kinase 2, and one of its inhibitors.^22^ Furthermore, in another recent paper, we showed the effect of Ca^2+^ concentration differences on the stability of cutinase, a hydrolytic enzyme, by analyzing our MD trajectories using the fraction of native contacts, *Q* value, which matched well with our observations from experiments.^23^ Thus, the combination of using high temperature MD simulations, followed by analysis by *Q* value, should yield insight into the stability of proteins.

In this work, we chose five *V*_H_H (4idl, 4w70, 1mel, 5sv4, and 4tyu) and took the *V*_H_ (1fvc, 3b9v) from two engineered antibodies, which have a wide range of *T*_m_. Since *V*_H_H and *V*_H_ only consist of the heavy chain, the CDR‐H1, CDR‐H2, and CDR‐H3 loops are called the CDR1, CDR2, and CDR3 loops, respectively. We then applied MD simulations at multiple temperatures to estimate their relative stability and compared them to the experimental data. The root mean square deviation (RMSD) against the initial crystal structures and the *Q* value along the MD trajectories were compared to the *T*_m_ values. The *Q* values showed a good correlation with the *T*_m_, so the residues were classified by their hydrophobicity and size, and were subsequently analyzed by calculating their respective *Q* values. Per‐residue *Q* values were also analyzed to identify residues contributing to the instability. To demonstrate the applicability of our protocol in designing mutant structures with higher stability, we designed several virtual mutant structures using 4idl as a base, since it has the lowest *T*_m_ of our structures, and performed additional MD simulations at 400 K to investigate their thermal stability *in‐silico* as a case study.

## Results

A dataset consisting of seven sdAbs (*V*_H_H and *V*_H_) was assembled for this study, since they have a wide range of *T*_m_ values and good resolutions for each crystal structure, which are listed in Table 1, along with the ranges of the CDR loops as obtained from PyIgClassify.^24^ One of the structures (PDB ID 1fvc) consists of both *V*_H_ and *V*_L_ in the crystal structure, while another one (PDB ID 1mel) includes its target antigen lysozyme in the crystal. In these cases, simulations and analysis were performed on the *V*_H_ and *V*_H_H, respectively. Figure 1 shows the alignment of their sequences (A) and the superposition of the structures (B). While the sequences in the framework are similar, those in the CDR region are diverse, especially the CDR3 region. We prepared seven computational systems consisting of the antibodies in explicit water at physiological ion concentration (4idl, 1fvc, 4w70, 1mel, 5sv4, 3b9v, and 4tyu). High temperature MD at 400 K and 500 K was executed with simulations at 300 K as a control. Ten 100 ns simulations using different random seeds for the initial velocities of the atoms were carried out for each antibody and temperature to increase statistics, with a total simulation time of 21 μs (100 ns × 7 antibodies × 3 temperatures × 10 random seeds).

In Figure S1, the average RMSD of the backbone over the final 30 ns with the standard deviation is plotted against the experimentally determined *T*_m_ of each sdAb. Both the average and the deviation become larger as the simulation temperature increases. While the structure fluctuates around that of the crystal structure at 300 K, it undergoes large changes at 500 K. The RMSD values are in between the two at 400 K, indicating the occurrence of moderate structural changes. However, at each simulation temperature, no significant difference of the average RMSDs between the antibodies is observed, suggesting that the RMSD is not a good measurement to indicate *T*_m_.

**Figure 2:**
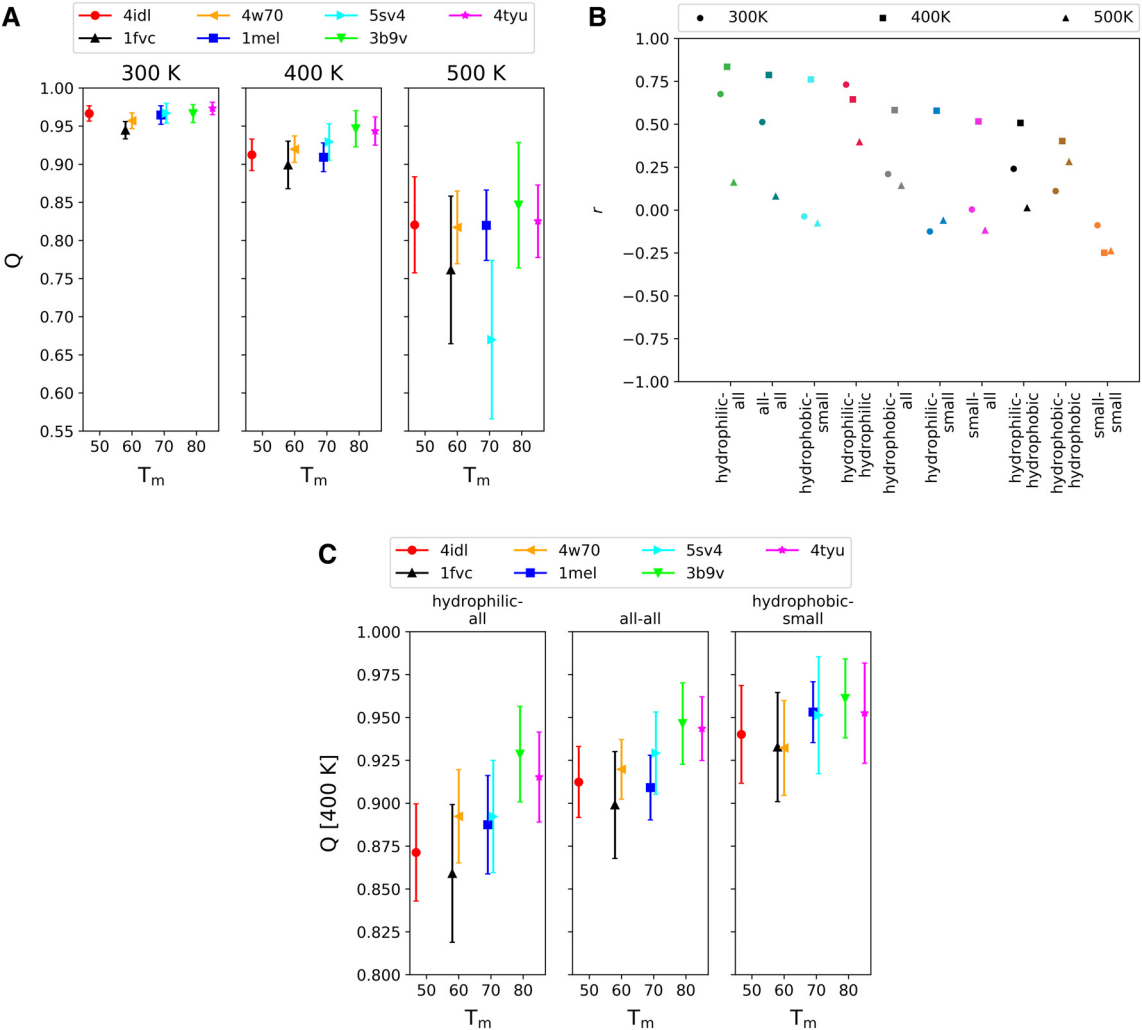
(A) Average *Q* value over the final 30 ns with standard deviation against the experimental *T*_m_ per simulation temperature (300 K, 400 K, and 500 K). The data for 4idl, 1fvc, 4w70, 1mel, 5sv4, 3b9v, and 4tyu are shown in red circle, black upper triangle, orange leftward triangle, blue square, cyan rightward triangle, green lower triangle, and magenta star, respectively, with error bars. (B) Pearson correlation coefficient (*r*) of each average *Q* value of the described group pairs to the experimental *T*_m_ for different group combinations and temperatures (300 K, 400 K, and 500 K). The hydrophilic‐all group is the average *Q* value between the hydrophilic residues (Asp, Glu, Gln, Asn, Arg, Lys, and His) versus all residues, the all–all group is the regular average *Q* value and the hydrophobic‐small group is the average *Q* value between the hydrophobic (Phe, Tyr, Trp, Leu, Val, Ile, Met, Cys, and Pro) versus the small (Gly, Ala, Ser, and Thr) residues. (C) Average *Q* value over the final 30 ns with standard deviation against the experimental *T*_m_ per group pair [top 3 in Fig. 2(B)] for the 400 K simulations.

Next, the *Q* value, which is a measurement of the degree of protein unfolding, was calculated in order to determine whether it correlates well with the *T*_m_. In Figure 2(A), the average *Q* value over the final 30 ns and the standard deviation are plotted against the experimentally determined *T*_m_ for each sdAb. The average *Q* values become smaller and the deviations become larger as the simulation temperature increases. The *Q* values range between 0.90 and 0.95 at 400 K. A weak correlation (Pearson correlation, *r* = 0.51) was found between the experimental *T*_m_ and the *Q* values at 300 K, which improved for the systems at 400 K (*r* = 0.79) and significantly diminished at 500 K (*r* = 0.08). Since our MD simulations at 400 K in combination with the *Q* value analysis appears to give a good measure of thermal stability of these sdAbs, we hereafter focus on the results obtained from the simulations at 400 K.

Figures S2–S8 show the backbone RMSD and the *Q* value along all trajectories at 400 K. For most of the trajectories, the RMSD increases by approximately 0.2 nm in the early stage of the simulation within 1 ns, after which the RMSD equilibrates out with the occasional slight drift. This observation is in contrast to those for the *Q* values, where the *Q* values for systems of low experimental *T*_m_ (4idl, 1fvc, 4w70, and 1mel) decreases by approximately 0.1, and continues to slowly decrease thereafter, while for the systems with a high experimental *T*_m_ (5sv4, 3b9v, and 4tyu), the *Q* value decreases much more slowly over time, usually taking 50 ns or longer to start dropping. These results suggest that the *Q* values over time can discriminate between thermally stable and unstable sdAbs.

We refine the stability measurement by analyzing the contribution of amino acid residue types by their property to the thermal stability. We classify them into three types; hydrophobic, hydrophilic, and small, as defined previously by Barthelemy et al.^12^ Figure 2(B) depicts the correlation coefficient between each average *Q* value obtained from the MD simulations at each temperature and the experimental *T*_m_ for several groups, where “all–all” corresponds to the pairs of all residues (i.e., considering all atomic contacts) and corresponds to the *Q* value in Figure 2(A). For most groups, the average *Q* value from the 400 K simulations exhibits a better correlation with the experimental *T*_m_ than that of the other temperatures. The *Q* value of the hydrophilic‐all pairs shows the best correlation coefficient, followed by the pairs of all–all, hydrophobic‐small, etc. In Figure 2(C), the average *Q* values with the standard deviation including the hydrophilic residue types are plotted against the experimental *T*_m_. In the hydrophilic‐all pair that showed the best correlation with *r* = 0.84, the average *Q* value of 1fvc could be classified as an outlier. The second highest correlated pair, all–all, shows a similar tendency to the highest pair and has a correlation of *r* = 0.79, where 1fvc and 1mel might be classified as outliers. The third highest correlated pair, hydrophobic‐small, has a correlation of *r* = 0.76. The three groups have increasingly higher *Q* values as their correlation decreases, with hydrophilic‐all having the lowest average *Q* value of the three.

Continuing our efforts to fine‐grain stability measurements, we investigated the contribution of each amino acid residue to the thermal stability by calculating the *Q* value of each individual residue from the MD simulations at 400 K and identifying the unstable residues (Tables S1–S7 and Figs. S12–S18). Here, we defined unstable residues as those with an average *Q* value lower than 0.6 measured during the final 30 ns of the simulation, i.e., residues that have lost their native contacts with respect to the X‐ray structure. The tables list the unstable residues detected for each structure, with those belonging to CDR loops (Table 1) annotated with a star symbol (*). Residues that have fewer than 30 atomic contacts (and thus a less reliable *Q* value) in the native structure are shown below each table and defined as the excluded residues. In Table S1, 16 unstable residues of 4idl are listed as well as the corresponding trajectory identifier and the average *Q* value. The N‐terminal residue and its neighbors (Lys1–Gln3), residues from CDR1 (Ala24–Ile31), CDR3 (Phe102, Trp107, Tyr110, and Glu111) and residues from a non‐CDR region (Phe62, Arg71, and Asn76) were identified as unstable residues. For 1fvc, 17 unstable residues were observed (Table S2), which are located near the N‐terminal (Val2 and Leu4), CDR1 (Ser25 and Phe27), CDR3 (Ser97‐Gly100, Phe104, Tyr105, and Met107‐Tyr109), and non‐CDR regions (Gln13 and Trp110‐Gln112). It is to be noted that two Gln residues (Gln13 and 112) appear especially unstable, where most native contacts are lost in 10 out of 10 and 9 out of 10 trajectories, respectively. Like 1fvc, 17 unstable residues were observed for 4w70 (Table S3). These residues were located near the N‐terminal (Val2, Gln3), CDR1 (Ala24, Ser25, and Arg27‐Phe29), CDR2 (Ala53, Ala54, Tyr59, and Tyr60), and non‐CDR regions (Ala14, Gly15, Ala75, Asn80, Lys90, and Pro91). The structure of 1mel (Table S4) is even more stable than those described above, with only 13 observed unstable residues. The unstable residues are located near the N‐terminal (Val2 and Gln3), CDR1 (Ser25, Tyr 27, and Tyr32), CDR3 (Ile102, Tyr103, Tyr107, Glu108, and His111), and non‐CDR regions (Arg45, Gly117, and Gly126). The structure of 5sv4 also has 13 unstable residues (Table S5), which are located near the N‐terminal (Val2), CDR1 (Arg27 and Tyr32), CDR2 (Arg53), CDR3 (Val98, Ile99, Thr106‐Arg108, Ser110, and Asp112), and non‐CDR regions (Gln13 and Phe47). The structure of 3b9v is much more stable (Table S6), where only seven unstable residues were observed near the N‐terminal (Val2), CDR1 (Phe27), CDR3 (Arg98 and Tyr105‐Met107), and a non‐CDR region (Trp110). The structure of 4tyu is also quite stable (Table S7), although 13 unstable residues were observed near CDR1 (Ser25, Arg27, Thr28, Phe29, and Arg31), CDR2 (Ala54, Tyr59), CDR3 (Tyr110), and non‐CDR regions (Gln13, Ala14, Gly15, Trp119, and Ser128). Most of the identified unstable residues however only have one or two trajectories affected. The *F*_v_ region (which is mostly conserved) is fundamentally stable, where the few unstable residues are mostly caused by either (i) being part of the N/C terminal, (ii) near unstable residues part of the CDR region (either by sequence or spatially), or (iii) near small residues (either sequentially or spatially). Since their local environments are unstable, some of this instability is translated to the nearby residues part of the *F*_v_. In all cases, the β‐sandwich motif does not unfold during the MD simulations at 400 K.

To demonstrate how our method could be used to identify potentially stabilizing mutants, we prepared a case study. Using the aforementioned information, we set out to improve the thermal stability of 4idl *in silico* based on the data obtained from Table S1 and Figure S12 and the structure of 4idl. We identified two mutations that we thought would lead to an increase in thermal stability. As shown in Figure S12, one is located in a non‐CDR loop (R71I). The other one is located in CDR1 (N27D), although this mutation could potentially decrease the specificity. To investigate the effects in terms of our *Q* value analysis, we prepared three virtual mutants, two of which are single mutants (R71I and N27D) and the third being the combination of the two. We performed simulations using the same protocol, but only at 400 K. Figures S9, S10, and S11 show the RMSD and *Q* values along the trajectories, for the R71I and N27D and the double mutant, respectively, where Table 2 shows the average RMSD and *Q* values obtained from the simulations’ final 30 ns. The R71I mutant is more stable than the wild type with 13 unstable residues (Table S8 and Fig. S19). The unstable residues were observed near the N‐terminal residue and its neighbors (Lys1‐Gln3), CDR1 (Ala24, Ser25, Asn27, Thr28, Ser30, and Ile31), CDR3 (Tyr110 and Glu111), and a non‐CDR region (Phe62, Asn76). For the N27D mutant 12 unstable residues were observed (Table S9 and Fig. S20) near the N‐terminal residue and its neighbors (Lys1‐Gln3), CDR1 (Ala24, Ser25, Thr28, and Ile31), CDR3 (Tyr110), and non‐CDR regions (Phe62, Arg71, Asn76, and Trp113). For the N27D/R71I double mutant, only nine unstable residues were observed (Table S10 and Figure S21) near the N‐terminal residue and its neighbor (Lys1 and Val2), CDR1 (Thr28 and Ser30), CDR3 (Phe102 and Trp107), and near non‐CDR regions (Arg45, Phe62, and Trp113). Both single mutations slightly increased the stability with respect to the *Q* value over the wild type and the double mutant even more (Table 2), to the point where it rivals the stability of 3b9v and 4tyu, sdAbs which have *T*_m_ values of around 80°C.

**Table 2:** 4idl Mutant Case Study Stability Statistics with Respect to the Wild Type

Computational system	RMSD (nm)	*Q*: hydrophilic‐all	*Q*: all–all	*Q*: hydrophobic‐small
4idl WT	0.20 (0.04)	0.87 (0.03)	0.91 (0.02)	0.94 (0.03)
R71I mutant	0.21 (0.06)	0.89 (0.03)	0.93 (0.02)	0.95 (0.03)
N27D mutant	0.16 (0.04)	0.89 (0.03)	0.93 (0.02)	0.97 (0.03)
Double mutant	0.15 (0.03)	0.92 (0.02)	0.95 (0.01)	0.97 (0.02)

*Note*: Comparison of the 4idl wild type system and our engineered virtual mutants as used in our case study. Shown are the measured average RMSD, average *Q* of hydrophilic‐all, all–all and hydrophobic‐small pairs with their standard deviations in parenthesis.

## Discussion

Our analysis protocol using MD trajectories obtained a fairly good correlation with the experimental *T*_m_ values [Fig. 2(B)], especially those for the *Q* values calculated from the hydrophilic‐all group and the all‐all group; however, there appear to be two outliers, i.e., 1fvc and 1mel in Figure 2(C). In the case of 1fvc, the original crystal structure consisted of both a *V*_H_ and a *V*_L_, while in the case of 1mel, the original crystal structure consisted of a *V*_H_ and an antigen. The removal of the *V*_L_ and antigen for 1fvc and 1mel, respectively, has most likely a negative effect on the stability with the respect to the crystal structure. The conformation of an sdAb can change upon binding to its target antigen, as recently shown by Garza et al.^25^ Since our analysis depends on the initial crystal structure, using an isolated sdAb for our analysis would be preferred.

In order to identify the unstable residues, we calculated a per‐residue *Q* value. The correlation coefficient of the average *Q* value over the residues of each system at 400 K with respect to the corresponding experimental *T*_m_ values, shows a relatively high correlation (*r* = 0.85, *Q* value data not shown), which suggests that the residues identified by our protocol should be representative of those that significantly contribute to the instability of an sdAb. To demonstrate the applicability of our protocol, we used the data obtained from the per‐residue analysis to identify two potentially stabilizing mutations: one in a non‐CDR loop and the other in CDR1. The analysis of the 400 K trajectories of the virtual mutant structures showed an improvement in the *Q* value and the combination of the two point mutations in the double mutant suggests that the improvements are additive (Table 2). We have demonstrated in our case study how our method can be used to identify unstable residues and generate potentially stabilizing mutants that have been validated *in silico* in terms of our analysis. However, after designing mutants using our method, in vitro validation would still be required in order to confirm their effective stability. In contrast the method by Barthelemy et al.,^12^ who improved their sdAb by replacing hydrophobic residues in the CDR3 loop interacting with the *V*_L_, we achieved similar improvements by making two mutations determined through studying the local interactions of the unstable residues identified by our per‐residue *Q* value analysis. While the approaches differed, both methods reached the same conclusion, which is that the correct placement of hydrophilic residues, or hydrophobic ones for that matter, leads to a better *T*_m_ value.

We have analyzed the Q value from the MD trajectories and found a correlation between the *Q* value and the *T*_m_. Whereas the *T*_m_ value measures the temperature at which half of the protein population is folded, while the other half is unfolded, the *Q* value measures the fluctuation of the protein in terms of the atomistic interactions. Recently, Katava et al. investigated the relationship between structural fluctuations and thermal unfolding and found that fast dynamics, i.e., local sub‐nanosecond timescale structural fluctuations, contributes significantly to the conformational entropy, affecting the thermal stability of proteins.^26^ Furthermore, they suggested that a certain amount of structural fluctuations triggers protein thermal unfolding. Our analysis using the *Q* value can give insight into which residues affect the local instability and thereby design mutants that mitigate this. Subsequently, this improvement in local instability should reduce the conformational entropy and reduce the total structural fluctuations, preventing the protein from triggering thermal unfolding. From this point of view, our analysis can provide useful insight into overall protein stability and to what degree potential mutants could affect the thermal stability.

Barthelemy et al.^12^ also described several mutants without determining their structure and primarily focused on mutations of Trp47, where mutations to medium sized hydrophobic residues improved the stability, while mutations to smaller hydrophobic or into hydrophilic residues reduced the stability. This result is in consistent with the local structure of 3b9v around Trp47, where smaller hydrophobic residues might be a better fit into the sub‐pocket where Trp47 sits, while hydrophilic residues would not have any potential bonding partners. When looking at our per‐residue *Q* value for Trp47, we observed an average value of 0.81 (± 0.06), suggesting that Trp47 is quite stable and mutations to the latter smaller or hydrophilic residues would have some negative impact on the *T*_m_. On overall, Barthelemy et al.^12^ described mostly mutants that have a minor impact on the *T*_m_, except for one double mutant, W47T/G35H (*T*_m_ = 65°C). Whereas W47T had a small negative impact on the *T*_m_ (75°C), G35H had a small positive impact on the *T*_m_ (80°C), suggesting that the combination of the two causes a disturbance of the local interaction network. In order to study the effect of this double mutation, we built a virtual double mutant of W47T/G35H and additionally changed the rotamer of W99 to prevent it from clashing with G35H. We ran simulations at 400 K, analyzed the resulting trajectories and found an average *Q* value of 0.88 (± 0.03), with the detailed plot of the trajectories shown in Figure S22. Our results show that the double mutant is significantly more unstable with respect to the wild type, which had a *Q* value of 0.95 (± 0.02), in agreement with their experimental data.

*Q* values calculated from hydrophilic residues showed a high correlation with the experimental *T*_m_ values [*r* = 0.84 for hydrophilic‐all in Fig. 2(B)]. When looking at the unstable residue tables (Tables S1–S7) over the seven systems, we can see that 30.5% corresponds to hydrophilic residues, 42.1% to hydrophobic residues and 27.4% corresponds to small residues, where these percentages were weighted by the number of trajectories affected per residue. However, to understand the high correlation, we need to compare the contribution of unstable hydrophilic residues per system. Doing so, we see that 47.2%, 39.7%, 21.5%, 11.8%, 39.2%, 4.5%, and 28.1% are hydrophilic for 4idl, 1fvc, 4w70, 1mel, 5sv4, 3b9v, and 4tyu, respectively. Looking at the relative stability of hydrophilic residues, we can see that 86.9%, 86.8%, 91.5%, 97.1%, 91.7%, 99.4%, and 95.7% is stable for 4idl, 1fvc, 4w70, 1mel, 5sv4, 3b9v, and 4tyu, respectively, ignoring the excluded residues. These data suggest that stabilization is somewhat correlated with favorable interactions to hydrophilic residues.

We performed MD simulations at 300 K, 400 K and 500 K. Our results suggest that 400 K is the most productive temperature, and as such we performed our mutation case study only at 400 K. Here, the higher temperature (i.e., 400 K) serves as a way to accelerate the dynamics without perturbing the structure, which happens at even higher temperatures, such as during our 500 K simulations. We believe that future work can focus only on the 400 K simulations, which will decrease the computational load and allow this method to be used in an industrial environment. On a small modern GPU cluster (10 GPUs), these simulations would take less than a day to complete for each mutant, enabling researchers to quickly obtain an idea about the relative stability of their sdAbs and how they could improve it. Since our results rely heavily on the crystal structure for calculating the *Q* value, an accurate model must first be produced if no experimentally determined structure is available. A potential strategy could be to first construct an initial model via an antibody modeling tool,^27^, ^28^ followed by an accurate, but computationally expensive sampling method such as multicanonical MD^18^, ^22^ to ensure the global minimum structure is found.

In this work, we have demonstrated our method using sdAbs, however our approach could potentially also be applied to a wide range of systems. For example, we previously employed high temperature simulations to quantitatively estimate the binding configuration of meta‐stable states between cyclin dependent kinase 2 and one of its inhibitors by using local, system‐specific reaction coordinates.^22^ However, we could instead use the *Q* value as a reaction coordinate, since *Q* values can be used as a general measurement for determining the stability of the binding configurations. As another example, our previous work on the dependency of the PET hydrolyzing enzyme cutinase to Ca^2+^ utilized a *Q* value based analysis procedure, but it was applied to investigate ion concentration dependencies as opposed to temperature dependencies. In short, we believe that our protocol has a wide range of applications for stability estimation, beyond the scope that we have described in this work and we intend to apply and refine our protocol and derivatives of it in our future works. As we have shown in this study that the results of our method correlate well with the thermal stability *T*_m_, in addition to our previously obtained tendencies of the relative stability of protein‐ligand complexes,^22^ we conclude that high‐temperature simulations in combination with *Q* value analysis can be an accurate and efficient method to estimate inter‐ and intra‐molecular stabilities *in‐silico*.

## Conclusion

We have described a protocol to use MD simulations in combination with *Q* value analysis to quantitatively predict the relative thermal stability of sdAbs showing a high correlation between the presence of hydrophilic residues and the experimental *T*_m_ value. Further analysis revealed that the number of unstable hydrophilic residues decreases as the *T*_m_ increases. Furthermore, we have demonstrated a case study exemplifying the use of our obtained data to drive the engineering of sdAbs to improve thermal stability by identifying unstable residues. The protocol described in our work can help focus the scope of experimental work by first identifying potentially stabilizing mutations, thus greatly reducing the time and costs involved with developing stabilizing mutants.

## Materials and Methods

The structures for 4idl, 1fvc, 4w70, 1mel, 5sv4, 3b9v, and 4tyu were obtained from Protein Data Bank Japan.^15^, ^16^ First, since 4idl has a missing C‐terminal loop, it was capped using NME (N‐methyl) and to keep the protocol consistent, all other systems were also NME‐capped. Gromacs 2016^29^ was used for the preparation of the computational systems and the execution of the MD simulations. Hydrogens were attached and a dodecahedron box surrounding each protein such that the edges of the box were at least 1.5 nm away from the protein was constructed and filled with water molecules and 0.1 M of NaCl. The Amber99‐sb‐ildn^30^, ^31^, ^32^ force field was used to parameterize the protein, TIP3P^33^ for the waters and Joung et al.’s monovalent ion parameters^34^ were used for the ions. Two steps of energy minimizations were performed, where for the first step position restraints on the heavy solute atoms were used, while for the second one, no restraints were applied. For each system and each temperature, 10 parallel simulations were performed, where for each simulation the initial velocities were initialized using a different random seed. First, a 100 ps NVT simulation at 300 K was used to equilibrate the velocities, followed by a 100 ps NPT simulation, which was concluded by a 100 ps NVT simulation at the corresponding temperature. For these equilibration simulations, position restraints were used on the heavy solute atoms. For the production run, 100 ns of unrestrained NVT simulation was performed at the corresponding temperature. A cutoff of 1.2 nm was used in combination with the Zero‐Dipole method to treat the long‐range electrostatic interactions, which we have evaluated and tested extensively.^18^, ^22^, ^23^, ^35^, ^36^, ^37^, ^38^, ^39^, ^40^, ^41^, ^42^ A timestep of 2 fs was used in combination with LINCS^43^ and SETTLE^44^ constraints for the protein and waters, respectively. Bussi et al.’s thermostat^45^ was used to maintain the temperature and the Parrinello‐Rahman barostat^46^ at 1 bar was used during the NPT simulation. During the production run the structure was saved every 10 ps.

The above described protocol was used on every system. We first executed MD simulations for 4idl, 1fvc, 4w70, 1mel, 5sv4, 3b9v, and 4tyu at 300 K, 400 K, and 500 K. Afterwards, based on the analysis of our results, we prepared two mutant structures and one double mutant structure from 4idl and performed the same protocol, but only at 400 K. For our analysis, we calculated various *Q* values, as described by Best et al.^47^ We defined three groups; hydrophilic (Asp, Glu, Gln, Asn, Arg, Lys, and His), hydrophobic (Phe, Tyr, Trp, Leu, Val, Ile, Met, Cys, and Pro) and small (Gly, Ala, Ser, and Thr) and calculated the average inter‐ and intra‐group *Q* values as well as each group with respect to all other groups. We also calculated the average *Q* values of each individual residue with respect to all surrounding ones to identify unstable residues. For the averages, the final 30 ns of the simulations was used. The images were drawn using Molmil,^14^ a WebGL‐based molecular viewer developed by Protein Data Bank Japan.^15^, ^16^

## Conflicts of Interest

The authors declare that they have no conflict of interest with the contents of this article.

## Supporting information


**Appendix S1:** Supporting Information
