# Supplementary material for: Thermal stability of single‐domain antibodies estimated by molecular dynamics simulations
Source: Protein Sci. 2018 Dec 20;28(2):429–38. doi: 10.1002/pro.3546 (PMC6319760; doi:10.1002/pro.3546)
Supplement: Supplementary file 1 — Appendix S1: Supporting Information [file PRO-28-429-s001.pdf]

Supplemental data for

Thermal stability of single domain antibodies estimated by molecular dynamics simulations

(Table S1-S10 and Figure S1-S22)

Table S1: Residues of 4idl for each trajectory which have an average per-residue Q-value less than 0.6 at 400 K

Residue	Trajectory ID : Average Q-value									
**LYS1**	1:0.10	2:0.31	3:0.06	4:0.11	5:0.22	6:0.10	7:0.10	8:0.06	9:0.12	10:0.11
**VAL2**	1:0.27	3:0.05	4:0.34	6:0.37	7:0.27	8:0.03	9:0.22	10:0.21		
**GLN3**	3:0.58	4:0.46	6:0.43	7:0.31	8:0.42	9:0.04	10:0.20			
**ALA24***	9:0.34									
**SER25***	3:0.55	4:0.45	6:0.42	7:0.35	8:0.34	9:0.02	10:0.19			
**ASN27***	1:0.28	3:0.04	4:0.05	5:0.53	6:0.42	7:0.08	8:0.07	9:0.04	10:0.08	
**THR28***	3:0.02	4:0.11	6:0.35	7:0.33	8:0.55	9:0.45	10:0.19			
**SER30***	3:0.52	4:0.51								
**ILE31***	3:0.57	4:0.51	9:0.58							
**PHE62**	2:0.59	6:0.54	7:0.59	8:0.40	9:0.57					
**ARG71**	3:0.58	6:0.55	8:0.55	9:0.55	10:0.54					
**ASN76**	3:0.53	6:0.59								
**PHE102***	1:0.38									
**TRP107***	1:0.44									
**TYR110***	3:0.53	4:0.56	5:0.58							
**GLU111***	5:0.57									

Excluded: GLY8 GLY9 ALA11 GLN13 GLY16 GLY26* ALA29* ALA40 PRO41 GLY42 LYS43 GLN44 THR54* GLY55* ALA57* ASP61 LYS64 GLY65 ASN73 ALA74 ASN75 GLU88 ASP103* GLY104* ALA105* ARG106* GLU109* GLN115 GLN118 SER122 SER123 NME124

Table S2: Residues of 1fvc for each trajectory which have an average per-residue Q-value less than 0.6 at 400 K

Residue	Trajectory ID : Average Q-value									
**VAL2**	1:0.22	3:0.57	4:0.06	5:0.19	9:0.07					
**LEU4**	1:0.52									
**GLN13**	1:0.41	2:0.47	3:0.43	4:0.46	5:0.46	6:0.42	7:0.39	8:0.40	9:0.44	10:0.41
**SER25***	1:0.51	4:0.52	5:0.58	9:0.57						
**PHE27***	3:0.46									
**SER97***	1:0.55									
**ARG98***	1:0.39	2:0.57	4:0.44							
**TRP99***	2:0.53	4:0.48								
**GLY100***	1:0.18	2:0.31	3:0.42	4:0.12	7:0.33	8:0.37	10:0.43			
**PHE104***	1:0.25	2:0.44	3:0.07	4:0.09	7:0.01	8:0.02	10:0.05			
**TYR105***	1:0.16	2:0.07	3:0.25	7:0.32	8:0.31	10:0.38				
**MET107***	1:0.22	2:0.03	4:0.25							
**ASP108***	1:0.01	2:0.21	3:0.60	4:0.11	7:0.56	8:0.60	10:0.60			
**TYR109***	1:0.15	4:0.11	9:0.53							
**TRP110**	1:0.47	4:0.43	7:0.60							
**GLY111**	1:0.33									
**GLN112**	1:0.30	2:0.39	3:0.45	4:0.40	6:0.44	7:0.40	8:0.41	9:0.43	10:0.52	

Excluded: GLU1 GLY8 GLY16 GLY26* ASP31* ALA40 PRO41 GLY42 LYS43 GLY44 ASN55* GLY56* ASP62 SER63 GLY66 THR74 SER75 LYS76 SER85 ARG87 ALA88 GLU89 GLY101 ASP102 GLY103 ALA106 SER120 NME121

Table S3: Residues of 4w70 for each trajectory which have an average per-residue Q-value less than 0.6 at 400 K

**Residue**	**Trajectory ID : Average Q-value**									
**VAL2**	1:0.03	2:0.57	3:0.05	4:0.49	5:0.18	6:0.05	7:0.05	8:0.06	10:0.04	
**GLN3**	1:0.22	3:0.44								
**ALA14**	4:0.05	7:0.05								
**GLY15**	4:0.08	7:0.13								
**ALA24***	1:0.38									
**SER25***	1:0.19	3:0.38								
**ARG27***	1:0.00	2:0.41	3:0.00	4:0.12	5:0.03	6:0.00	7:0.04	8:0.05	9:0.58	10:0.02
**THR28***	1:0.31	2:0.24	3:0.48	4:0.54	6:0.26	7:0.55	8:0.08			
**PHE29***	1:0.04	2:0.55	3:0.54	4:0.14	5:0.15	6:0.57	7:0.47	10:0.46		
**ALA53***	2:0.27	6:0.48	7:0.39	8:0.43	9:0.37	10:0.25				
**ALA54***	1:0.35	2:0.33	6:0.31	7:0.08	8:0.56	9:0.07	10:0.19			
**TYR59***	2:0.31	6:0.31	7:0.60	9:0.48	10:0.53					
**TYR60***	2:0.57	7:0.59	8:0.60	9:0.60	10:0.57					
**ALA75**	6:0.59									
**ASN80**	2:0.58	3:0.59	8:0.53							
**LYS90**	4:0.49	7:0.50								
**PRO91**	4:0.51									

Excluded: GLU1 GLY8 GLY10 GLN13 ASP16 GLY26* SER30* ARG31* ALA32* ALA40 PRO41 GLY42 LYS43 GLU44 PRO55* GLY56* THR57* ALA58* ALA61* ASP65 SER66 ARG68 GLY69 SER77 ALA78 LYS79 GLU92 LYS104* GLN106* ASN112* ARG114* SER115* SER129 NME130

Table S4: Residues of 1mel for each trajectory which have an average per-residue Q-value less than 0.6 at 400 K

**Residue**	**Trajectory ID : Average Q-value**								
**VAL2**	1:0.33	2:0.47	3:0.15	5:0.46	8:0.53				
**GLN3**	3:0.56								
**SER25***	3:0.52	7:0.59	10:0.57						
**TYR27***	1:0.06	2:0.17	5:0.25						
**TYR32***	1:0.53	4:0.58	5:0.48	6:0.40	7:0.50	8:0.57	9:0.60		
**ARG45**	8:0.57								
**ILE102***	1:0.20	2:0.46	3:0.25	5:0.42	6:0.35	7:0.36	9:0.47		
**TYR103***	1:0.19	3:0.05	5:0.45	6:0.24	7:0.35	9:0.58			
**TYR107***	1:0.27	2:0.57	3:0.24	4:0.55	5:0.60	6:0.36	7:0.45	8:0.57	9:0.45
**GLU108***	3:0.12	10:0.51							
**HIS111***	3:0.31	10:0.50							
**GLY117**	3:0.57	7:0.16	10:0.51						
**GLY126**	9:0.33	10:0.57							

Excluded: ASP1 GLY8 GLY9 SER11 GLY16 GLY26* THR28* ILE29* GLY30* ALA40 PRO41 GLY42 LYS43 GLU44 GLY54* GLY55* ASP62 SER63 GLY66 ASN74 ALA75 LYS76 GLU87 PRO88 GLU89 THR101* ALA104* SER105* TYR106* SER114 THR115 GLY116 TYR118 GLY119 GLY124 GLN125 SER133 NME134

Table S5: Residues of 5sv4 for each trajectory which have an average per-residue Q-value less than 0.6 at 400 K

**Residue**	**Trajectory ID : Average Q-value**							
**VAL2**	3:0.35	6:0.53	8:0.49	9:0.04	10:0.08			
**GLN13**	1:0.59	2:0.57	3:0.48	5:0.57	6:0.48	8:0.58	9:0.60	10:0.48
**ARG27***	3:0.41	6:0.21	8:0.14	9:0.22	10:0.07			
**TYR32***	3:0.42	10:0.30						
**PHE47**	8:0.57							
**ARG53***	4:0.57							
**VAL98***	10:0.58							
**ILE99***	8:0.58							
**THR106***	7:0.34	8:0.19						
**SER107***	8:0.08							
**ARG108***	8:0.20							
**SER110***	1:0.58	6:0.47	7:0.44	8:0.27				
**ASP112***	8:0.41							

Excluded: GLU1 GLY8 ASP16 GLY26* THR28* GLY30* ASP31* ALA40 PRO41 GLY42 LYS43 GLU44 SER54* THR55* ILE56* ASP62 SER63 GLY66 SER74 ALA75 LYS76 PRO88 GLU89 PRO102* VAL103* TYR104* ALA105* ASN109* ASP111* GLN118 SER125 NME127

Table S6: Residues of 3b9v for each trajectory which have an average per-residue Q-value less than 0.6 at 400 K

**Residue**	**Trajectory ID : Average Q-value**							
**VAL2**	7:0.07							
**PHE27***	2:0.40	5:0.51	6:0.51	7:0.58	9:0.46	10:0.41		
**ARG98***	7:0.48							
**TYR105***	1:0.12	7:0.08						
**ALA106***	1:0.40	7:0.14						
**MET107***	1:0.59	7:0.14						
**TRP110**	1:0.39	2:0.52	3:0.22	4:0.34	7:0.30	8:0.52	9:0.47	10:0.60

Excluded: GLU1 GLY8 GLN13 GLY16 GLY26* ASN28* ASP31* ALA40 PRO41 GLY42 LYS43 GLY44 THR54* ASN55* GLY56* ASP62 SER63 GLY66 SER75 LYS76 ALA88 GLU89 GLY100* GLY101* ASP102* GLY103* PHE104* ASP108* TYR109 GLY111 GLN112 NME120

Table S7: Residues of 4tyu for each trajectory which have an average per-residue Q-value less than 0.6 at 400 K

**Residue**	**Trajectory ID : Average Q-value**						
**GLN13**	5:0.28	8:0.47					
**ALA14**	5:0.18	8:0.18					
**GLY15**	5:0.26	8:0.15					
**SER25***	6:0.55	7:0.54					
**ARG27***	2:0.57	4:0.23	6:0.60	7:0.00	8:0.58		
**THR28***	2:0.59	6:0.54	7:0.09				
**PHE29***	6:0.53	7:0.32					
**ARG31***	4:0.29	7:0.14					
**ALA54***	1:0.19	3:0.19	4:0.40	5:0.21	7:0.16	8:0.53	10:0.21
**TYR59***	4:0.10	7:0.55					
**TYR110***	4:0.45						
**TRP119**	1:0.57						
**SER128**	5:0.51						

Excluded: GLU1 VAL2 GLY8 ASP16 GLY26* SER30* ALA40 PRO41 GLY42 LYS43 GLU44 ALA53 PRO55* GLY56* THR57* ALA58* ALA61 ASP65 SER66 ARG68 GLY69 SER77 ALA78 LYS79 GLU92 LYS104* GLN106* ASN112* ARG114 SER115 GLN121 SER129 NME130

Table S8: Residues of 4idl R71I mutant for each trajectory which have an average per-residue Q-value less than 0.6 at 400 K

**Residue**	**Trajectory ID : Average Q-value**									
**LYS1**	1:0.24	2:0.10	3:0.05	4:0.06	5:0.22	6:0.21	7:0.38	8:0.26	9:0.13	10:0.08
**VAL2**	2:0.20	3:0.03	4:0.21	6:0.52	8:0.56	9:0.29	10:0.35			
**GLN3**	2:0.50	3:0.54	4:0.20	9:0.18	10:0.51					
**ALA24***	9:0.41									
**SER25***	2:0.47	3:0.46	4:0.05	9:0.08	10:0.56					
**ASN27***	2:0.23	3:0.06	4:0.18	5:0.53	8:0.44	9:0.05	10:0.12			
**THR28***	2:0.01	3:0.60	6:0.36	8:0.38	9:0.03	10:0.56				
**SER30***	2:0.55	4:0.49	9:0.55							
**ILE31***	2:0.54	4:0.53								
**PHE62**	3:0.60	5:0.51	6:0.56	9:0.46						
**ASN76**	2:0.54	9:0.18								
**TYR110***	2:0.60	4:0.57								
**GLU111***	9:0.55									

Excluded: GLY8 GLY9 ALA11 GLN13 GLY16 GLY26* ALA29* ALA40 PRO41 GLY42 LYS43 GLN44 THR54* GLY55* ALA57* ASP61 LYS64 GLY65 ASN73 ALA74 ASN75 GLU88 ASP103* GLY104* ALA105* ARG106* GLU109* GLN115 GLN118 SER122 SER123 NME124

Table S9: Residues of 4idl N27D mutant for each trajectory which have an average per-residue Q-values less than 0.6 at 400 K

Residue	Trajectory ID : Average Q-value									
**LYS1**	1:0.40	2:0.14	3:0.05	4:0.36	5:0.44	6:0.47	7:0.45	8:0.42	9:0.40	10:0.43
**VAL2**	2:0.19	3:0.03								
**GLN3**	2:0.05	3:0.35								
**ALA24***	2:0.56									
**SER25***	2:0.02	3:0.29								
**THR28***	2:0.08	3:0.27								
**ILE31***	2:0.57									
**PHE62**	1:0.56	3:0.52	4:0.48	6:0.45	9:0.54					
**ARG71**	2:0.53	3:0.53	4:0.49	5:0.56	7:0.57	10:0.48				
**ASN76**	2:0.52	3:0.58								
**TYR110***	2:0.54									
**TRP113**	7:0.45	10:0.60								

Excluded: GLY8 GLY9 ALA11 GLN13 GLY16 GLY26* ASP27* ALA29* ALA40 PRO41 GLY42 LYS43 GLN44 THR54* GLY55* ALA57* ASP61 LYS64 GLY65 ASN73 ALA74 ASN75 GLU88 ASP103* GLY104* ALA105* ARG106* GLU109* GLN115 GLN118 SER122 SER123 NME124

Table S10: Residues of 4idl R71I/N27D double mutant for each trajectory which have an average per-residue Q-value less than 0.6 at 400 K

Residue	Trajectory ID : Average Q-value									
**LYS1**	1:0.38	2:0.49	3:0.30	4:0.45	5:0.59	6:0.34	7:0.42	8:0.28	9:0.45	10:0.22
**VAL2**	10:0.54									
**THR28***	10:0.14									
**SER30***	10:0.57									
**ARG45**	9:0.57									
**PHE62**	1:0.55	3:0.46	6:0.58							
**PHE102***	5:0.60									
**TRP107***	5:0.56									
**TRP113**	9:0.46									

Excluded: GLY8 GLY9 ALA11 GLN13 GLY16 GLY26* ASP27* ALA29* ALA40 PRO41 GLY42 LYS43 GLN44 THR54* GLY55* ALA57* ASP61 LYS64 GLY65 ASN73 ALA74 ASN75 GLU88 ASP103* GLY104* ALA105* ARG106* GLU109* GLN115 GLN118 SER122 SER123 NME124

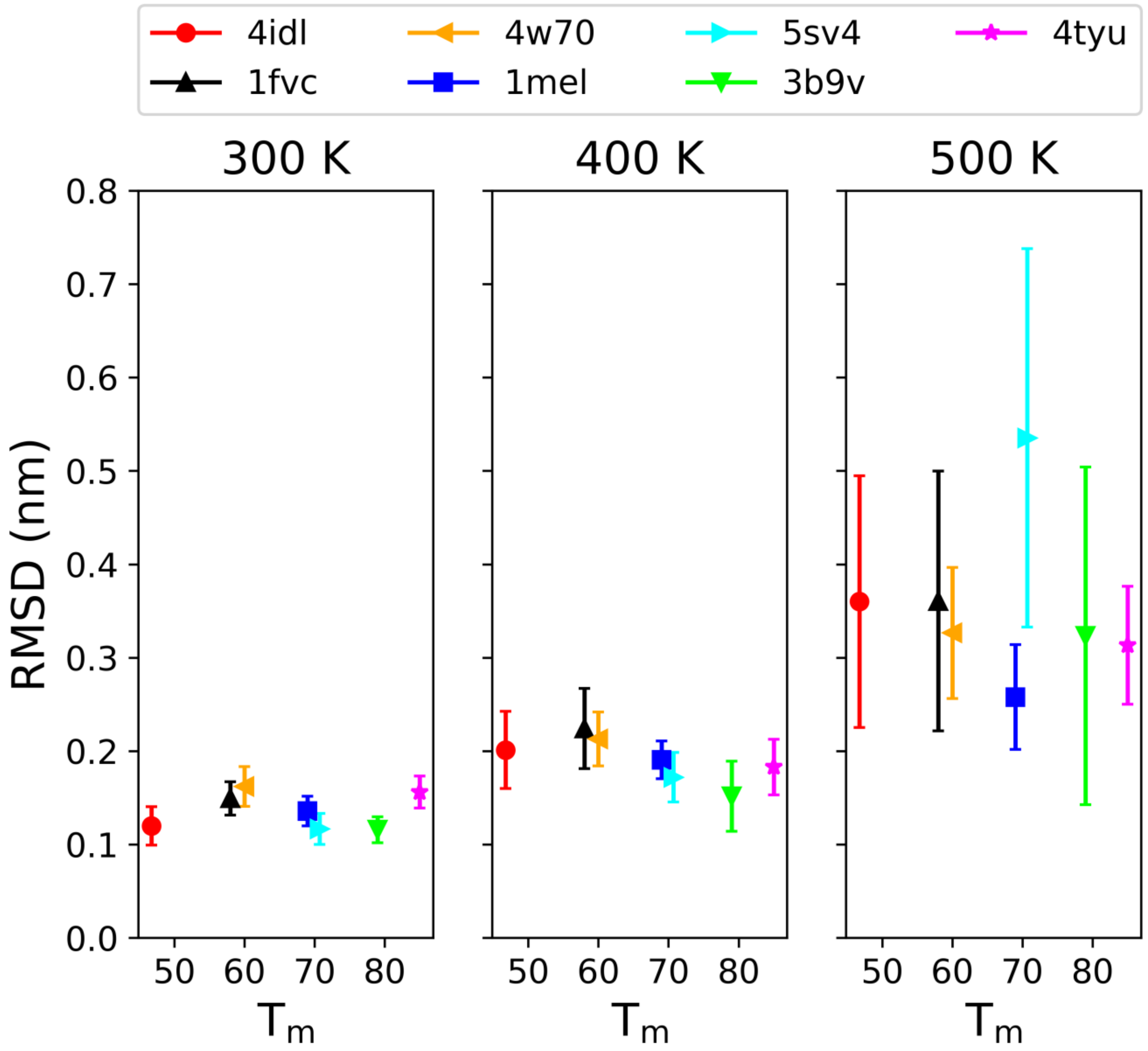

Figure S1: Average RMSD over the final 30 ns with standard deviation against the experimental T_m per simulation temperature (300 K, 400 K, 500 K). The data for 4idl, 1fvc, 4w70, 1mel, 5sv4, 3b9v and 4tyu are shown in red circle, black upper triangle, orange leftward triangle, blue square, cyan rightward triangle, green lower triangle and magenta star, respectively, with error bars.

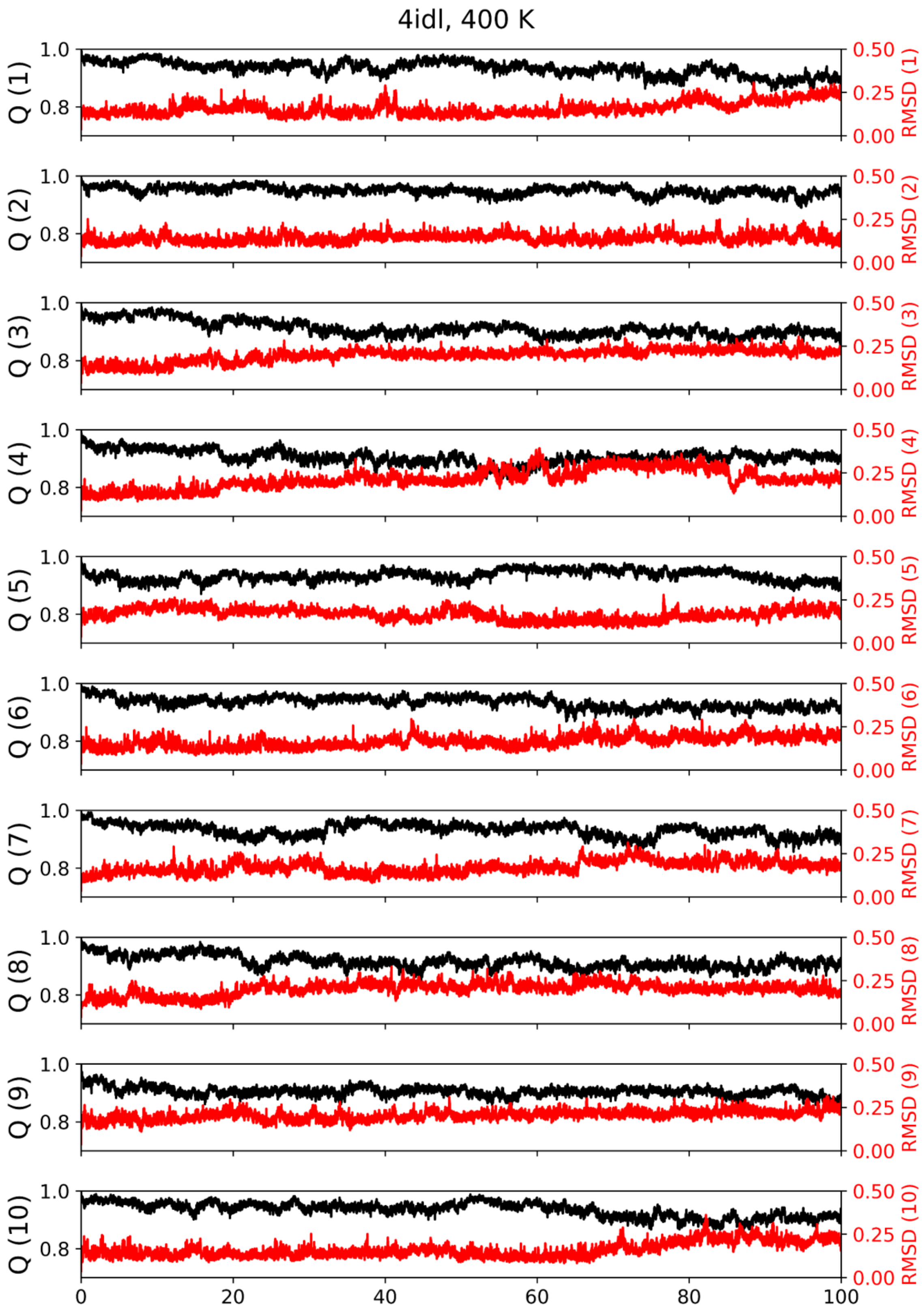

Figure S2: Q-value (black) and RMSD (red) versus time in ns along each parallel trajectory (Trajectory ID: 1-10) at 400 K for 4idl.

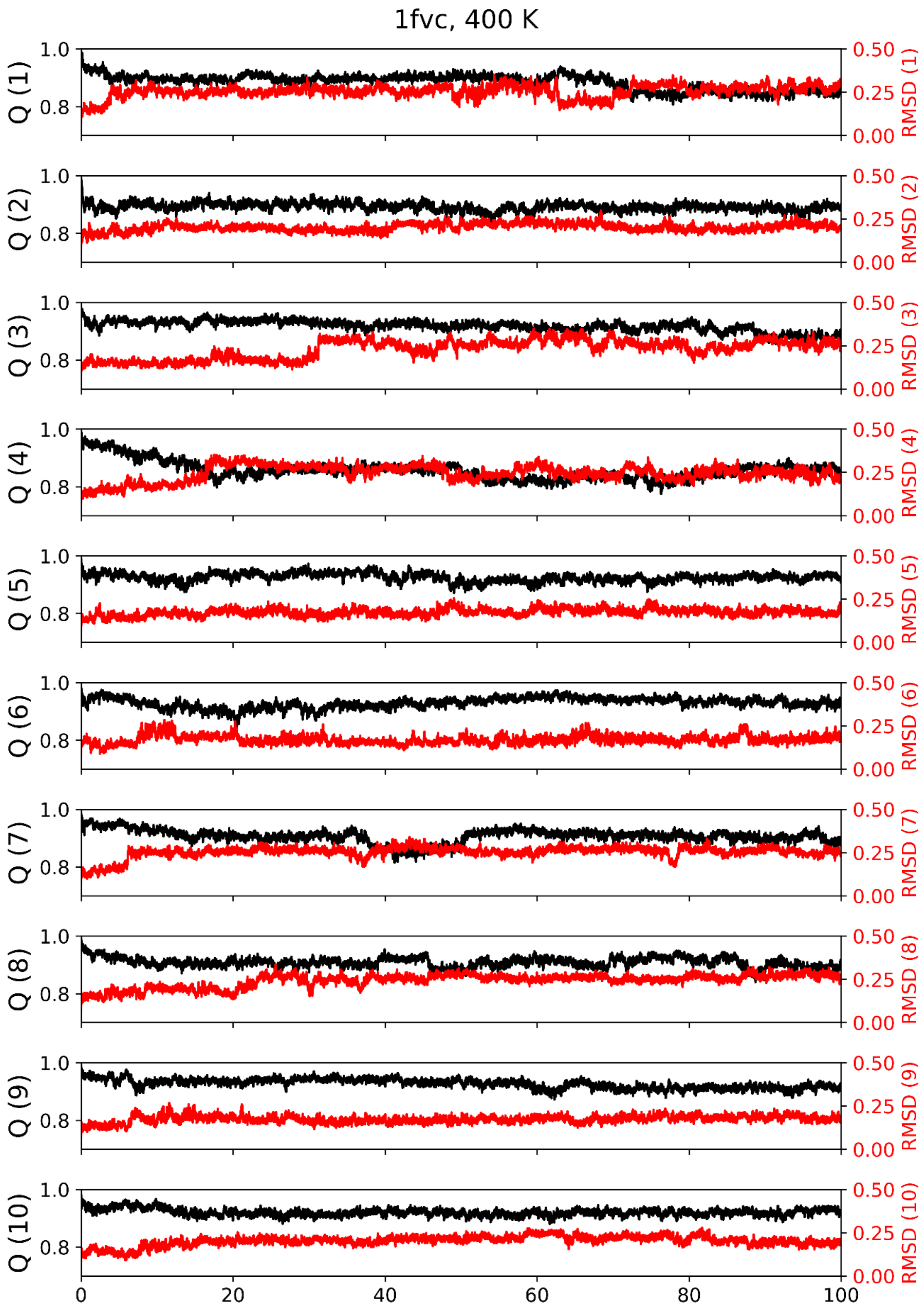

Figure S3: Q-value (black) and RMSD (red) versus time in ns along each parallel trajectory (Trajectory ID: 1-10) at 400 K for 1fvc.

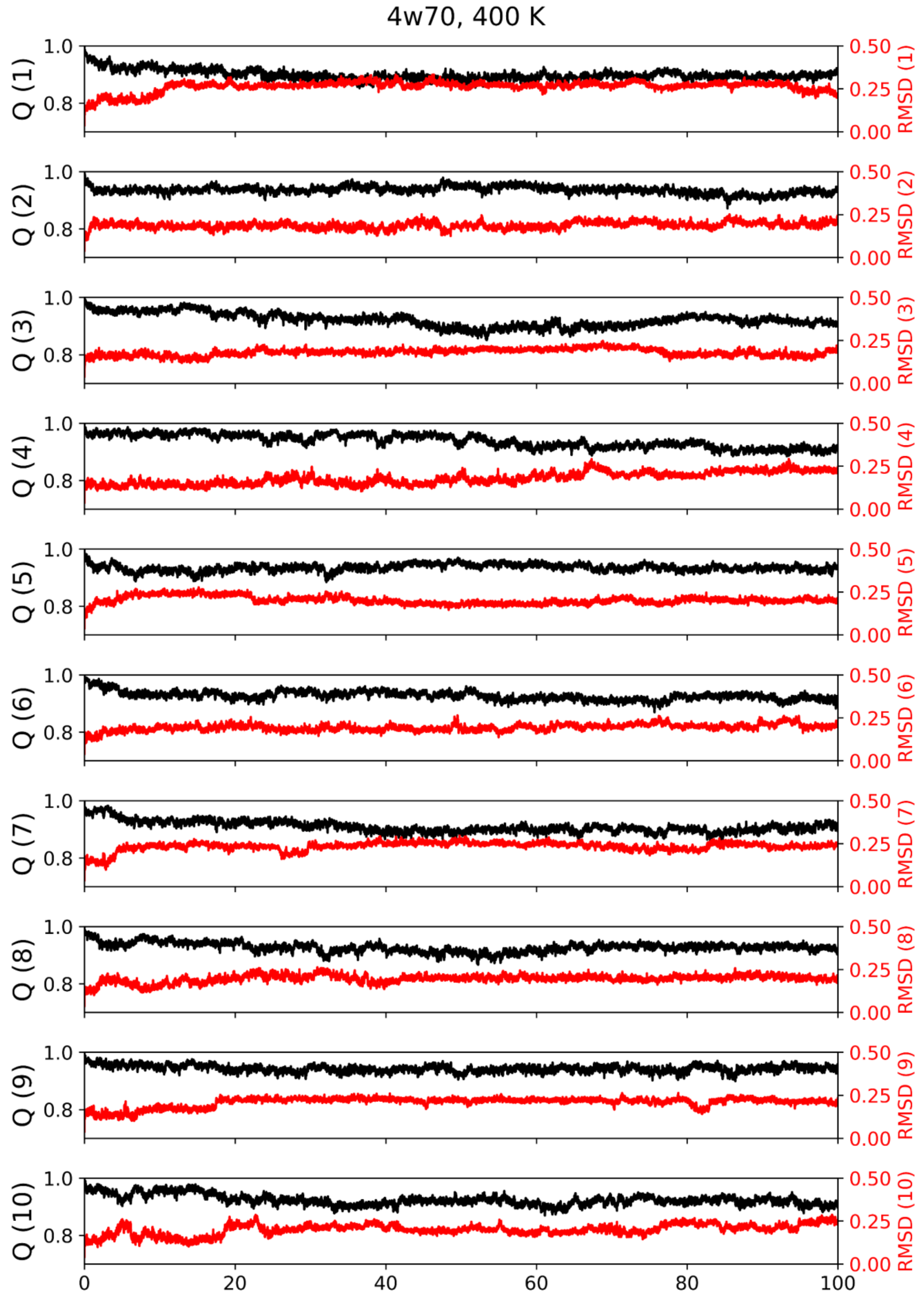

Figure S4: Q-value (black) and RMSD (red) versus time in ns along each parallel trajectory (Trajectory ID: 1-10) at 400 K for 4w70.

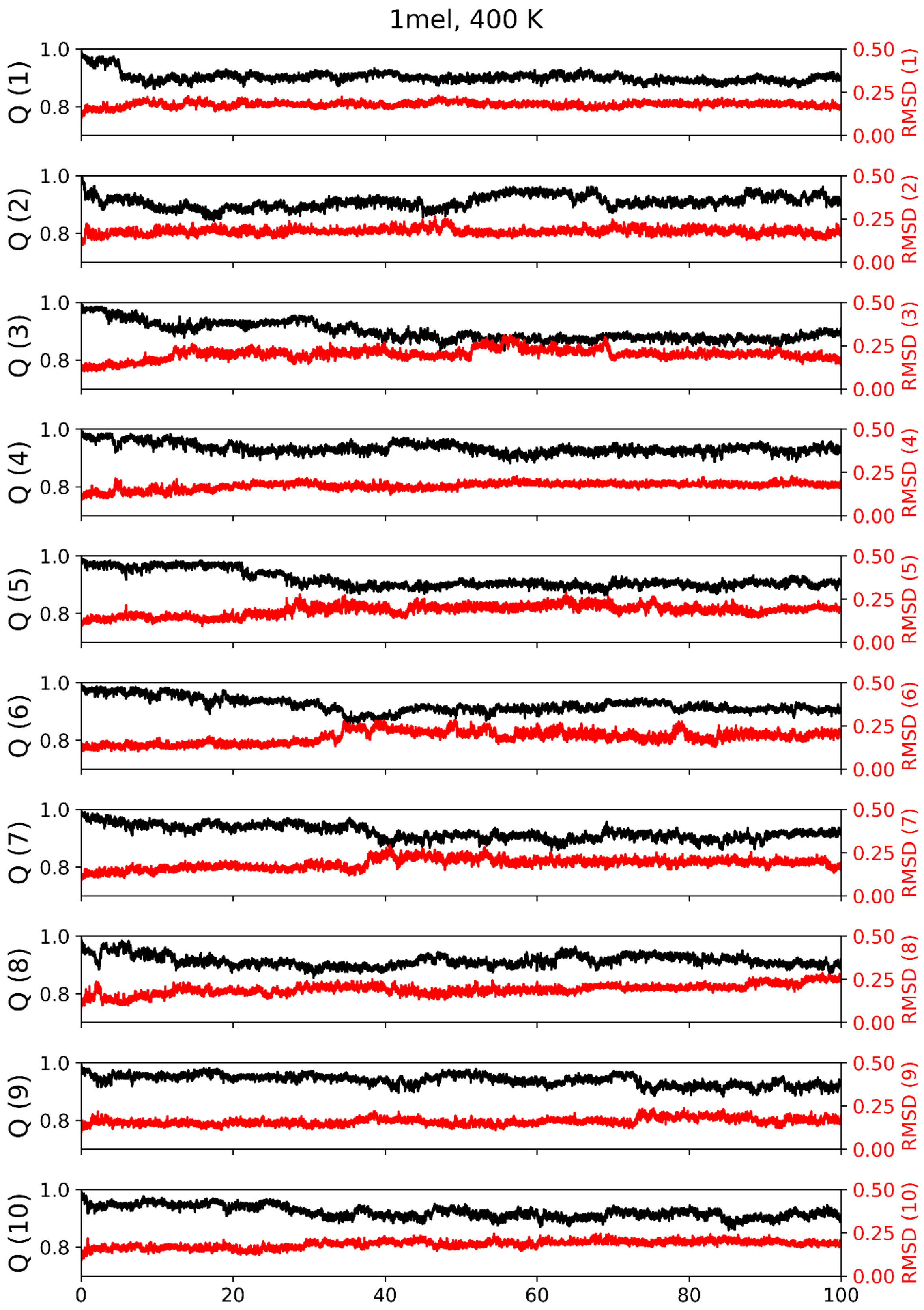

Figure S5: Q-value (black) and RMSD (red) versus time in ns along each parallel trajectory (Trajectory ID: 1-10) at 400 K for 1mel.

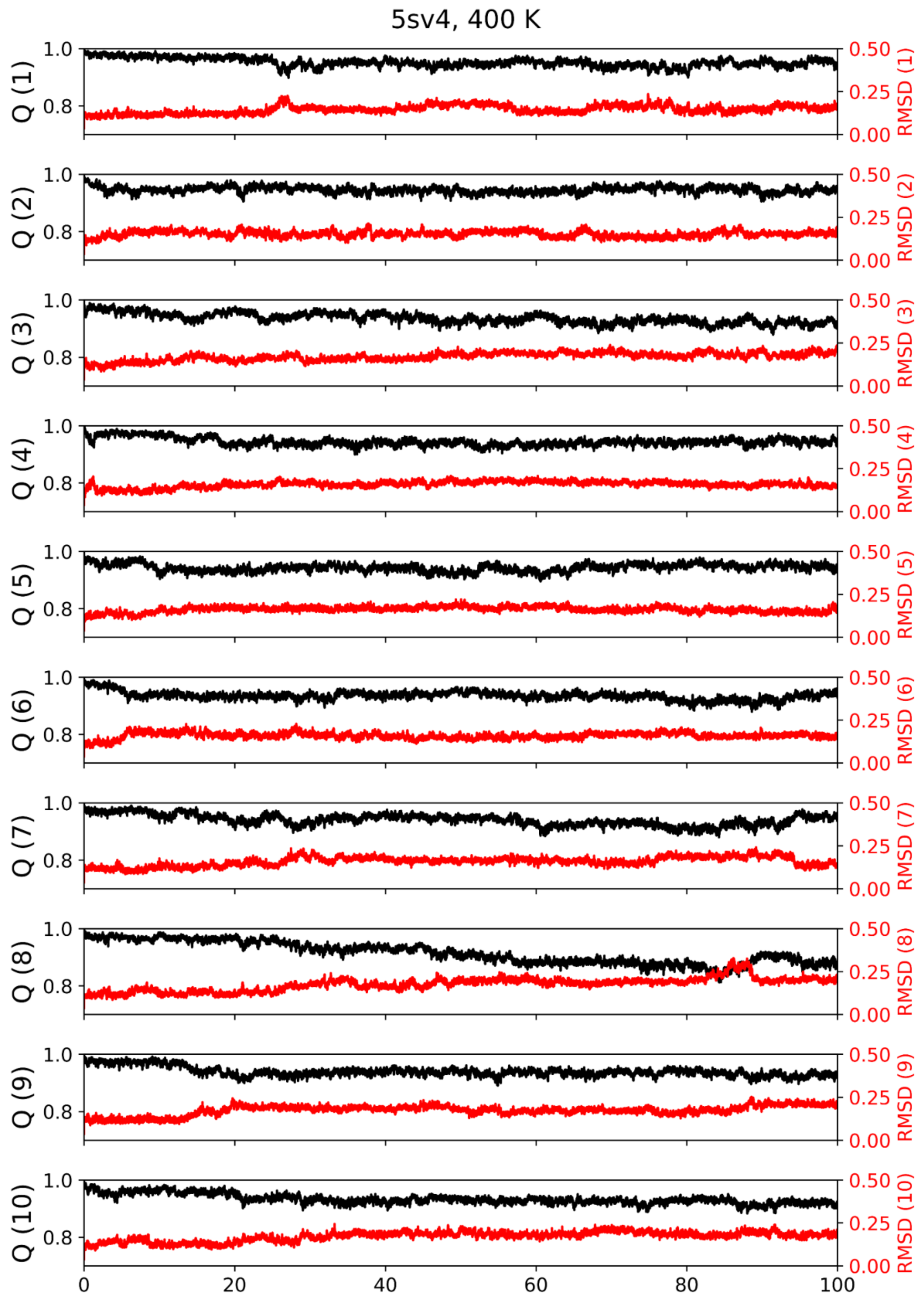

Figure S6: Q-value (black) and RMSD (red) versus time in ns along each parallel trajectory (Trajectory ID: 1-10) at 400 K for 5sv4.

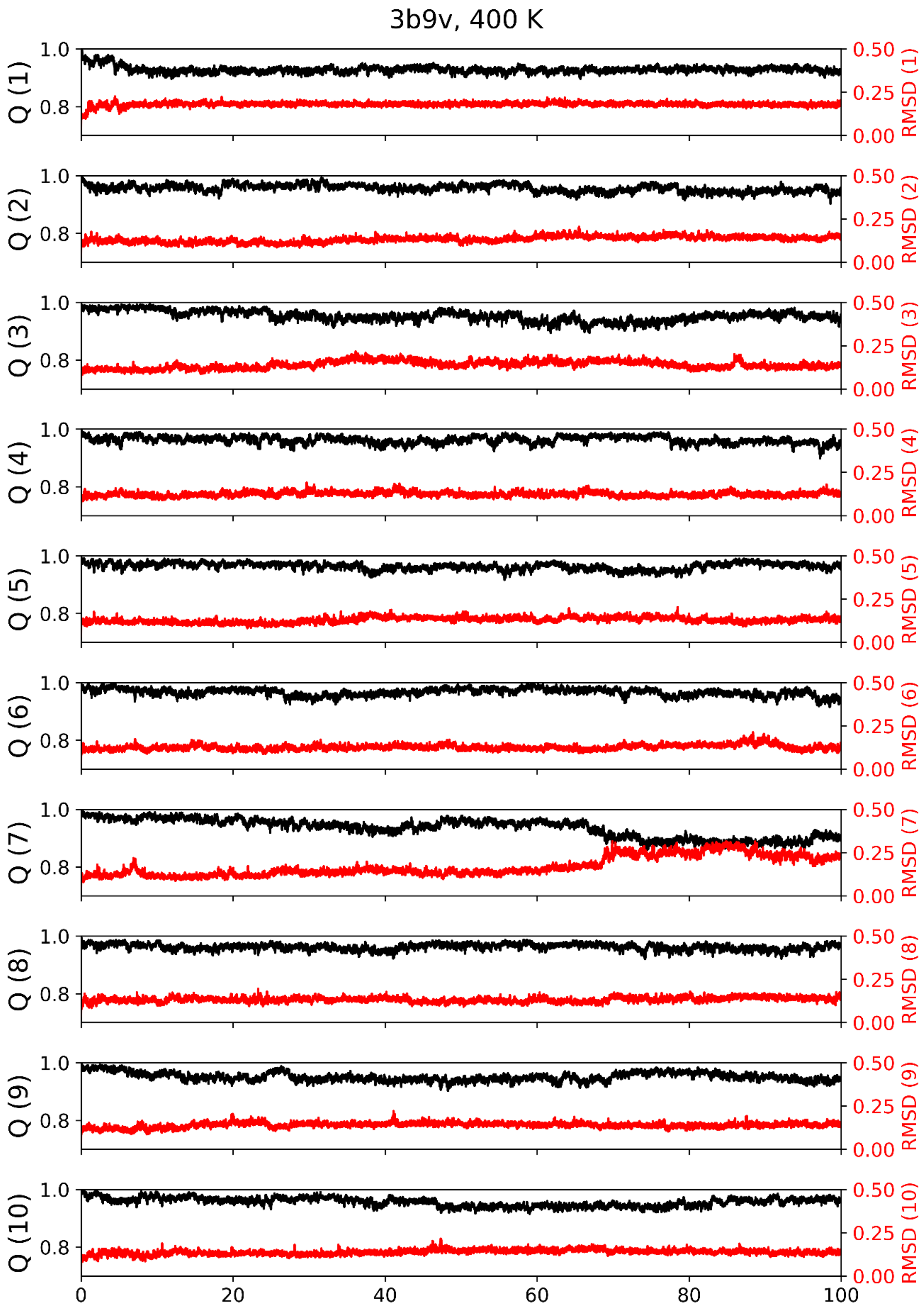

Figure S7: Q-value (black) and RMSD (red) versus time in ns along each parallel trajectory (Trajectory ID: 1-10) at 400 K for 3b9v.

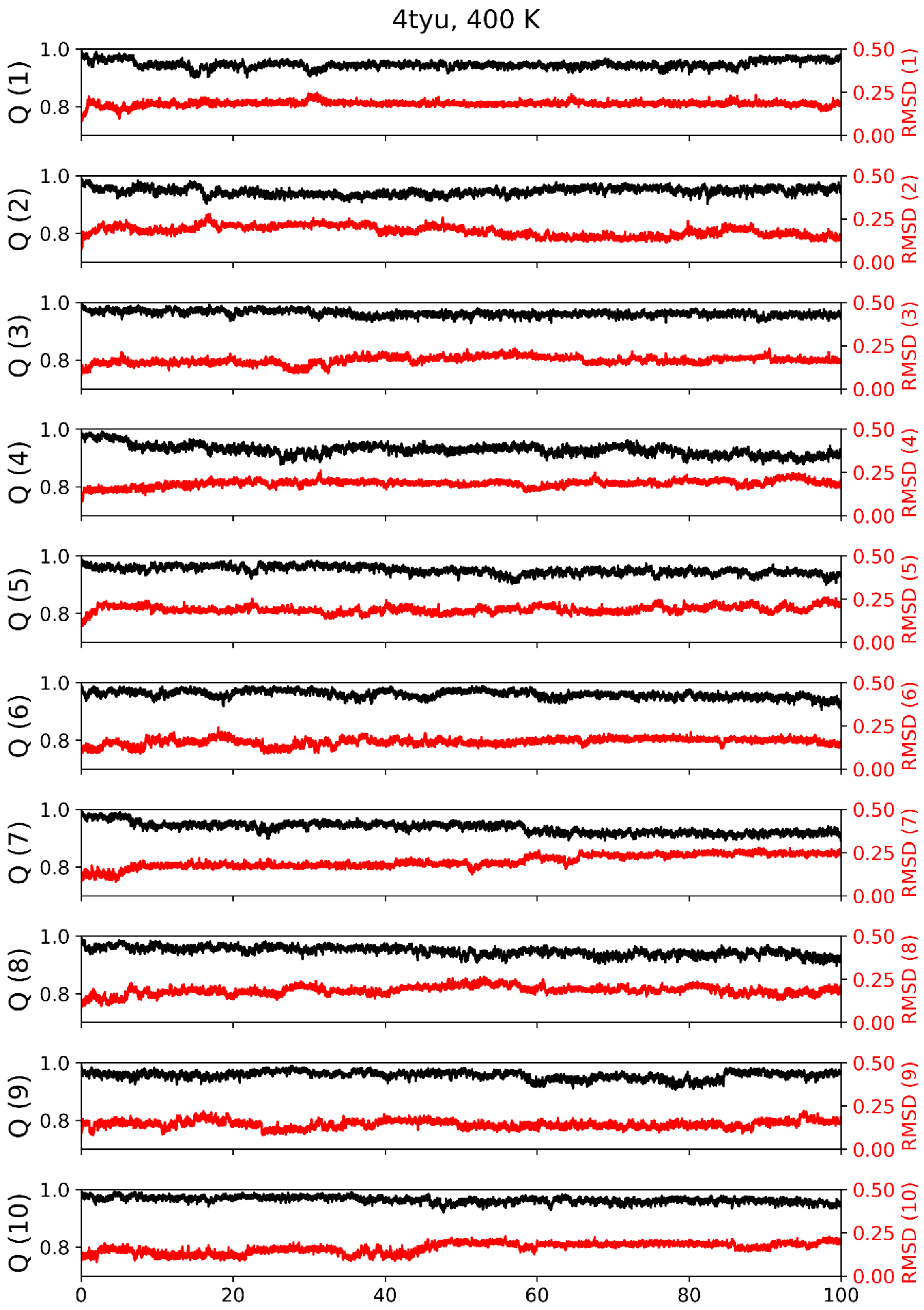

Figure S8: Q-value (black) and RMSD (red) versus time in ns along each parallel trajectory (Trajectory ID: 1-10) at 400 K for 4tyu.

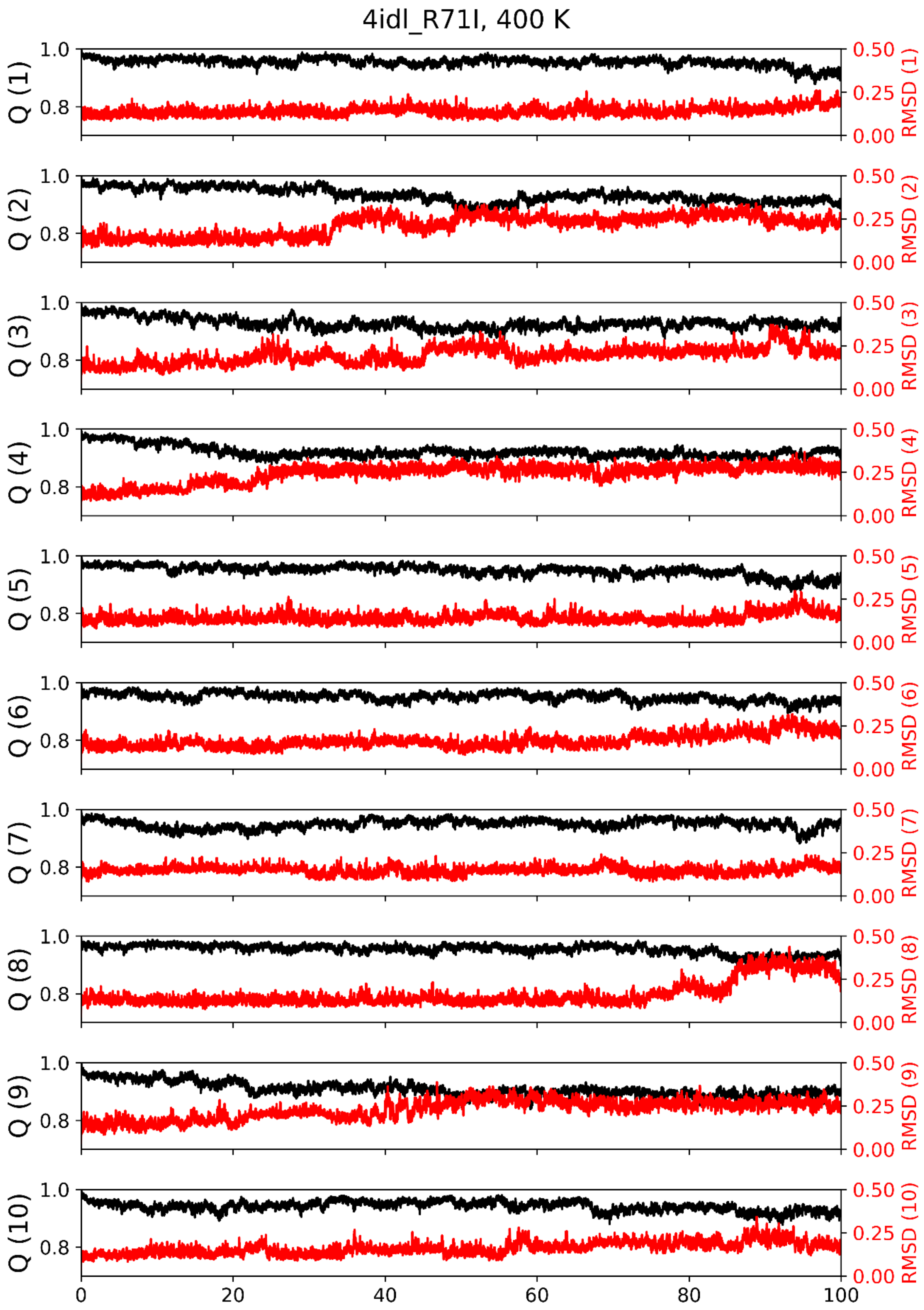

Figure S9: Q-value (black) and RMSD (red) versus time in ns along each parallel trajectory (Trajectory ID: 1-10) at 400 K the 4idl R71I mutant.

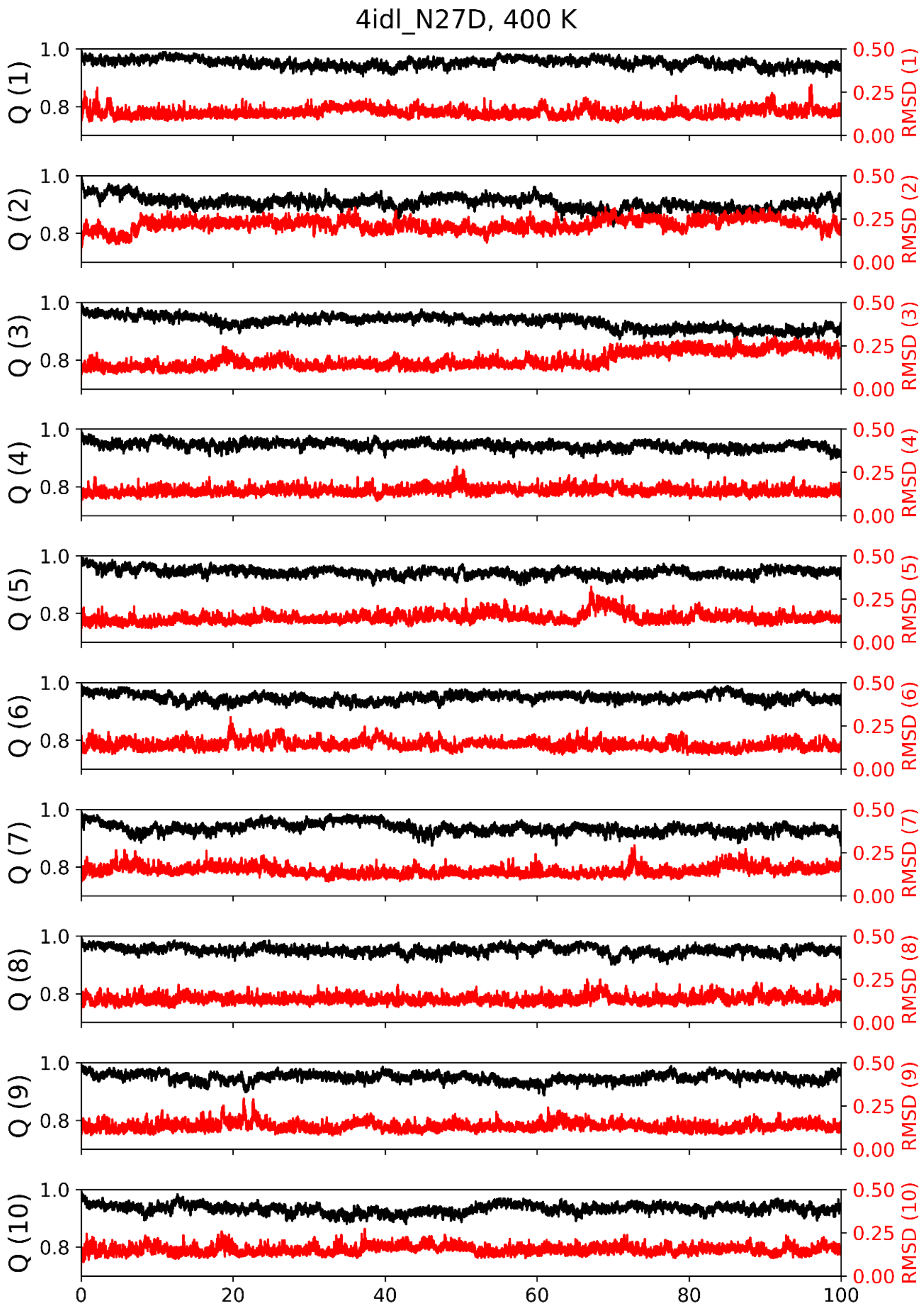

Figure S10: Q-value (black) and RMSD (red) versus time in ns along each parallel trajectory (Trajectory ID: 1-10) at 400 K the 4idl N27D mutant.

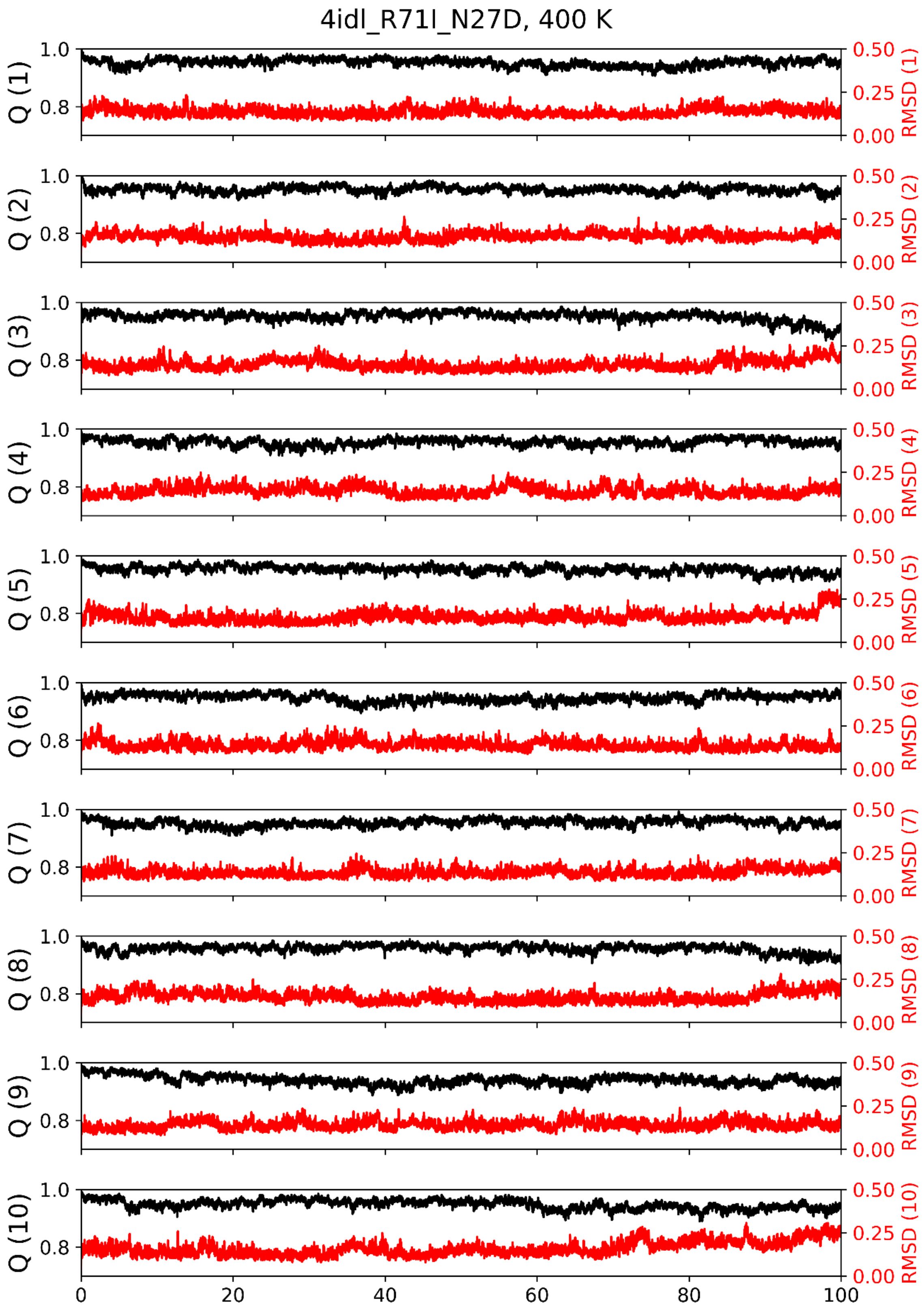

Figure S11: Q-value (black) and RMSD (red) versus time in ns along each parallel trajectory (Trajectory ID: 1-10) at 400 K the 4idl R71I/N27D mutant.

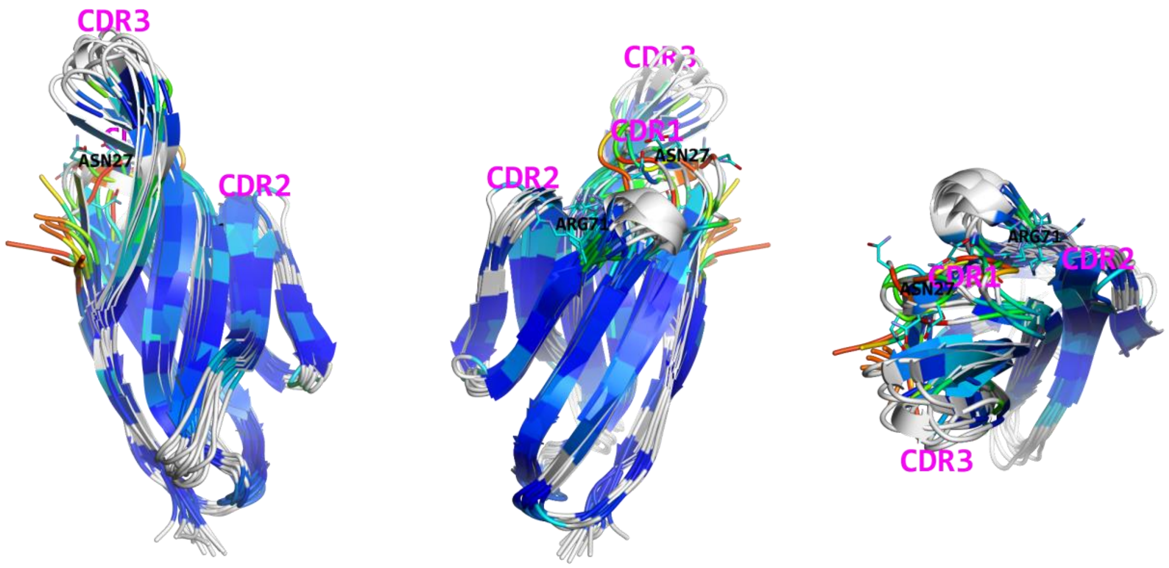

Front view Back view Top view

Figure S12: Average structures obtained during each of the 10 parallel trajectories at 400 K for 4idl colored by the average per-residue Q-value (0 to 1: red to blue), with excluded residues in white. The CDR regions are indicated in magenta denoted as CDR1, CDR2 and CDR3 and are placed at the top of each loop. The residues involved in our mutations (Asn27, Arg71) are shown, colored in cyan and denoted in black text.

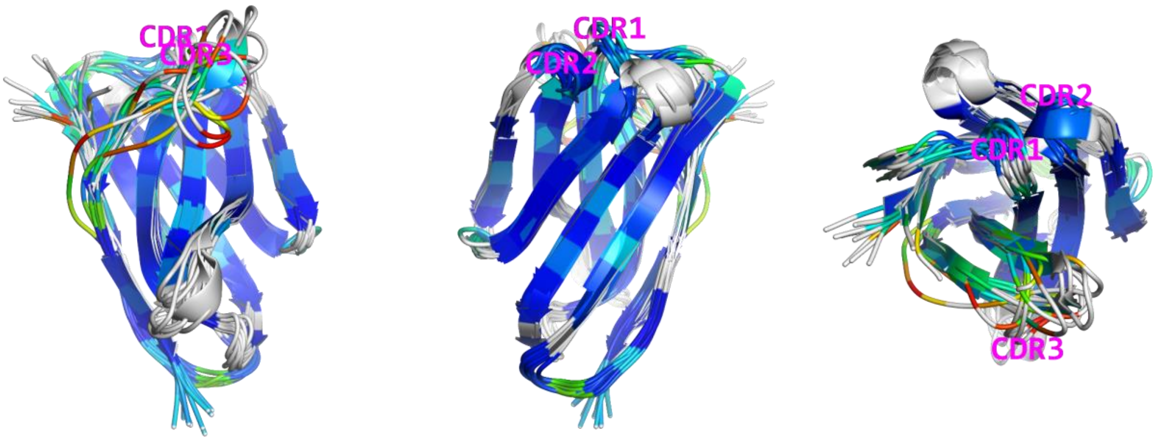

Front view Back view Top view

Figure S13: Average structures obtained during each of the 10 parallel trajectories at 400 K for 1fvc colored by the average per-residue Q-value (0 to 1: red to blue), with excluded residues in white. The CDR regions are indicated in magenta denoted as CDR1, CDR2 and CDR3 and are placed at the top of each loop.

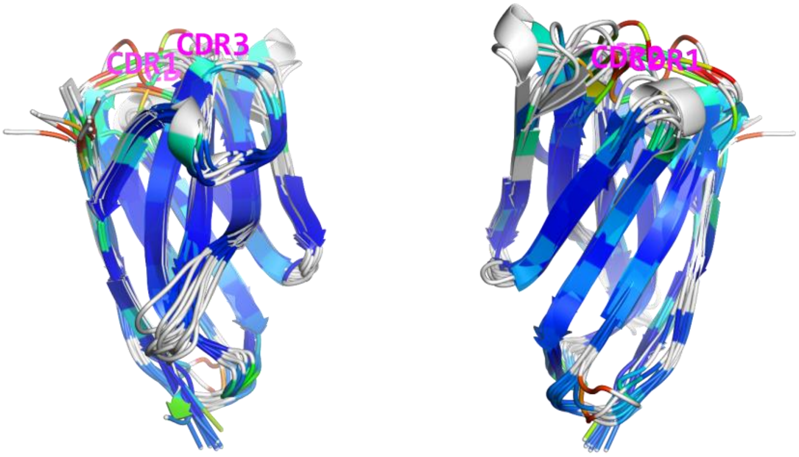

Front view Back view Top view

Figure S14: Average structures obtained during each of the 10 parallel trajectories at 400 K for 4w70 colored by the average per-residue Q-value (0 to 1: red to blue), with excluded residues in white. The CDR regions are indicated in magenta denoted as CDR1, CDR2 and CDR3 and are placed at the top of each loop.

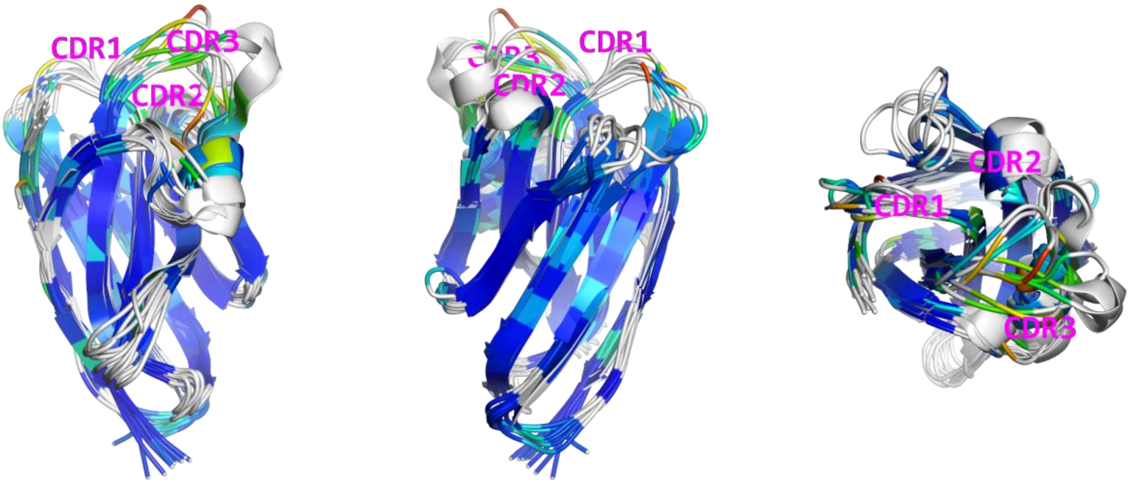

Front view Back view Top view

Figure S15: Average structures obtained during each of the 10 parallel trajectories at 400 K for 1mel colored by the average per-residue Q-value (0 to 1: red to blue), with excluded residues in white. The CDR regions are indicated in magenta denoted as CDR1, CDR2 and CDR3 and are placed at the top of each loop.

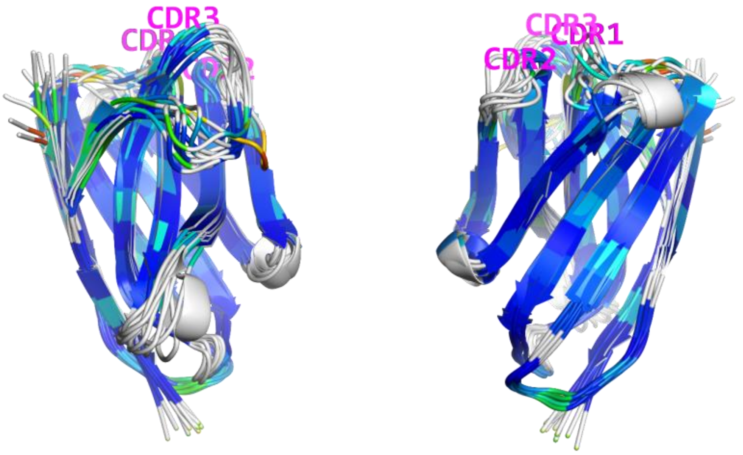

Front view Back view Top view

Figure S16: Average structures obtained during each of the 10 parallel trajectories at 400 K for 5sv4 colored by the average per-residue Q-value (0 to 1: red to blue), with excluded residues in white. The CDR regions are indicated in magenta denoted as CDR1, CDR2 and CDR3 and are placed at the top of each loop.

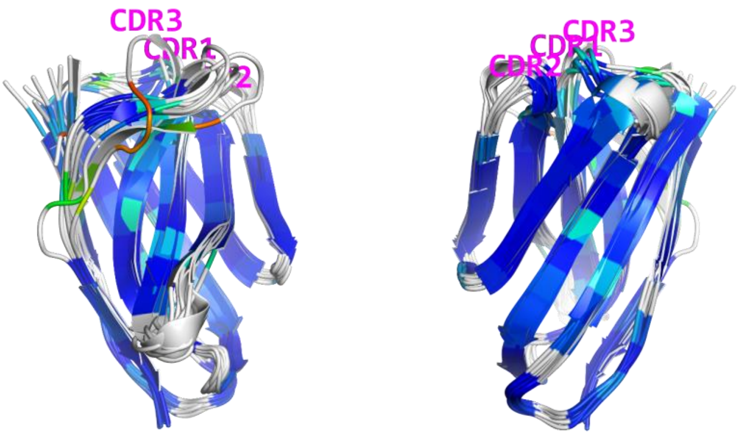

Front view Back view Top view

Figure S17: Average structures obtained during each of the 10 parallel trajectories at 400 K for 3b9v colored by the average per-residue Q-value (0 to 1: red to blue), with excluded residues in white. The CDR regions are indicated in magenta denoted as CDR1, CDR2 and CDR3 and are placed at the top of each loop.

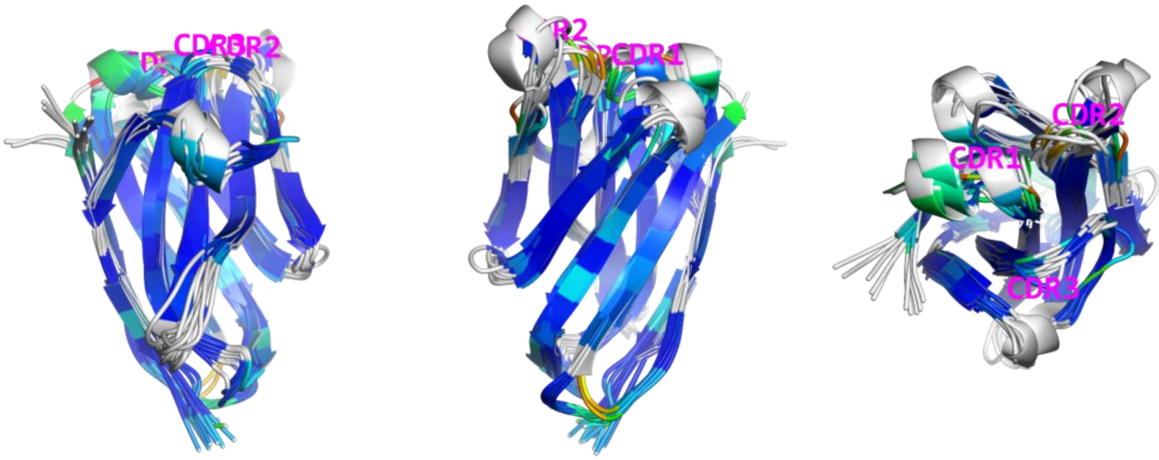

Front view Back view Top view

Figure S18: Average structures obtained during each of the 10 parallel trajectories at 400 K for 4tyu colored by the average per-residue Q-value (0 to 1: red to blue), with excluded residues in white. The CDR regions are indicated in magenta denoted as CDR1, CDR2 and CDR3 and are placed at the top of each loop.

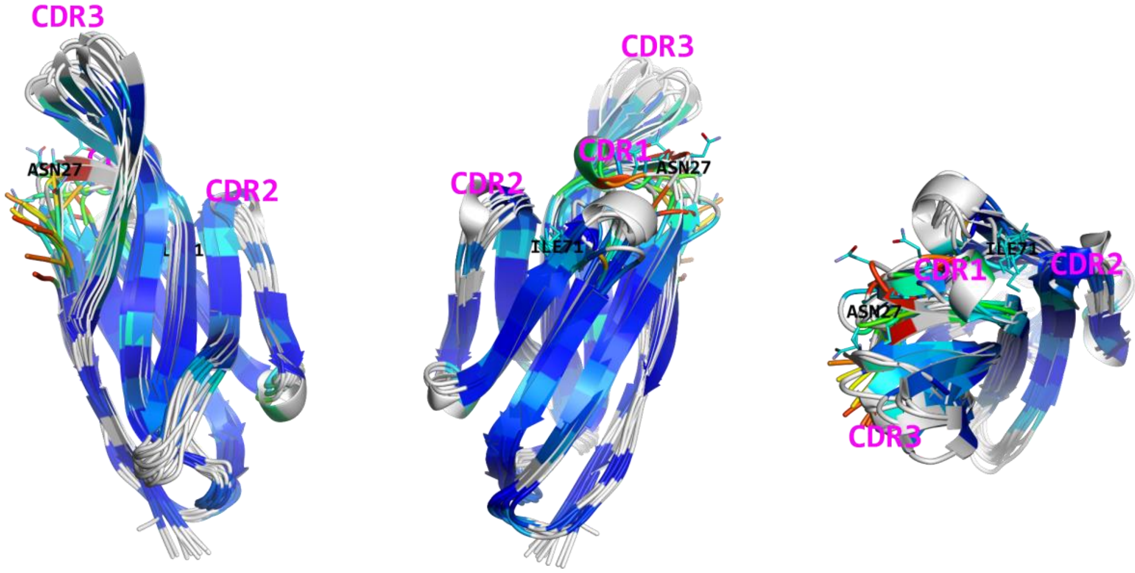

Front view Back view Top view

Figure S19: Average structures obtained during each of the 10 parallel trajectories at 400 K for the 4idl R71I mutant colored by the average per-residue Q-value (0 to 1: red to blue), with excluded residues in white. The CDR regions are indicated in magenta denoted as CDR1, CDR2 and CDR3 and are placed at the top of each loop. The residues involved in our mutations (Asn27, Ile71) are shown, colored in cyan and denoted in black text.

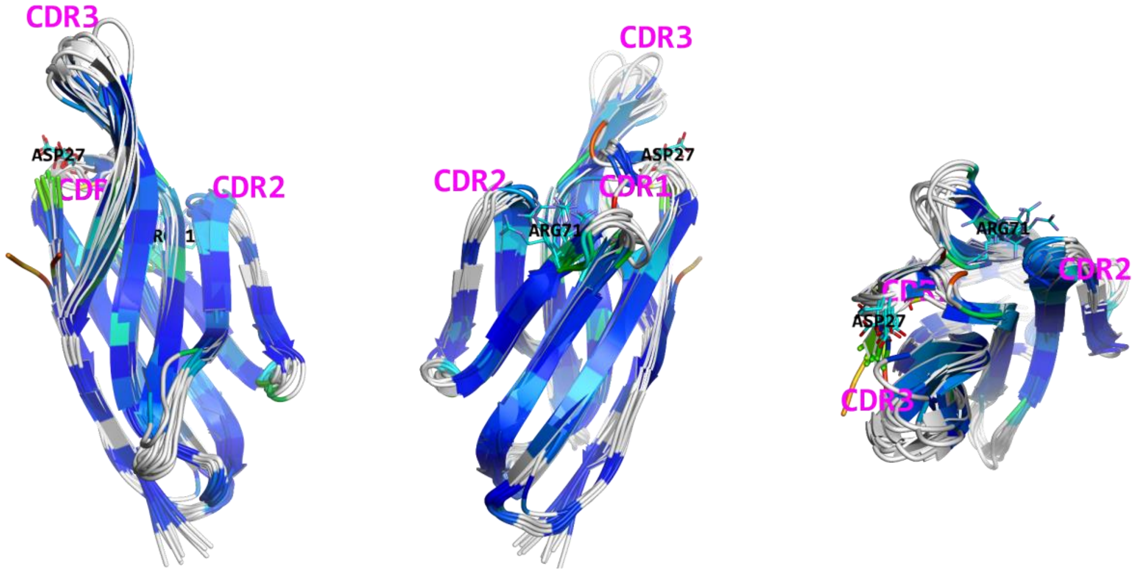

Front view Back view Top view

Figure S20: Average structures obtained during each of the 10 parallel trajectories at 400 K for the 4idl N27D mutant colored by the average per-residue Q-value (0 to 1: red to blue), with excluded residues in white. The CDR regions are indicated in magenta denoted as CDR1, CDR2 and CDR3 and are placed at the top of each loop. The residues involved in our mutations (Asp27, Arg71) are shown, colored in cyan and denoted in black text.

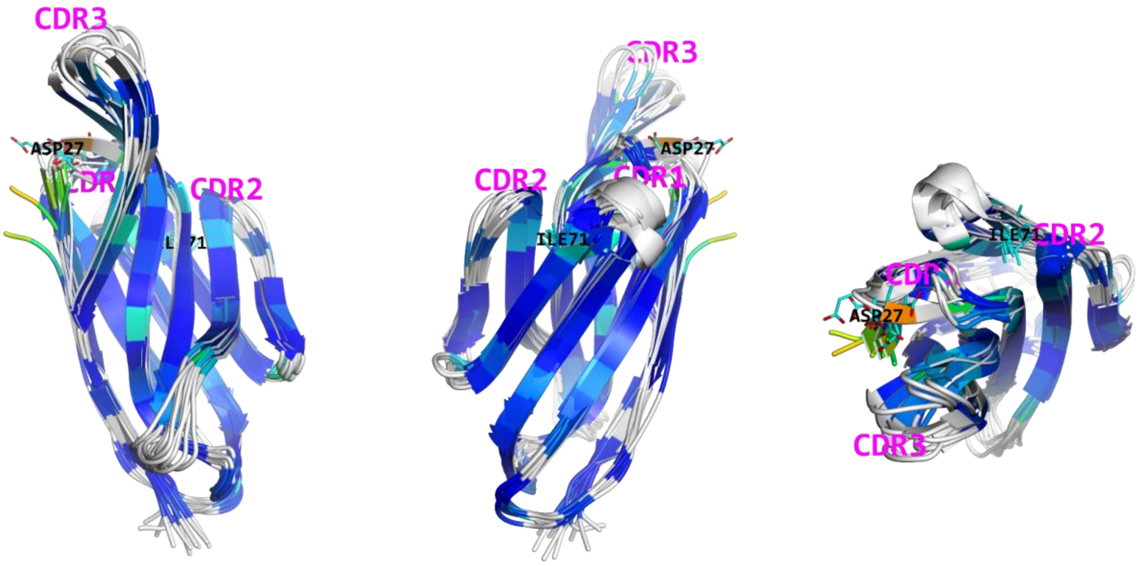

Front view Back view Top view

Figure S21: Average structures obtained during each of the 10 parallel trajectories at 400 K for the 4idl R71I/N27D mutant, respectively colored by the average per-residue Q-value (0 to 1: red to blue), with excluded residues in white. The CDR regions are indicated in magenta denoted as CDR1, CDR2 and CDR3 and are placed at the top of each loop. The residues involved in our mutations (Asp27, Ile71) are shown, colored in cyan and denoted in black text.

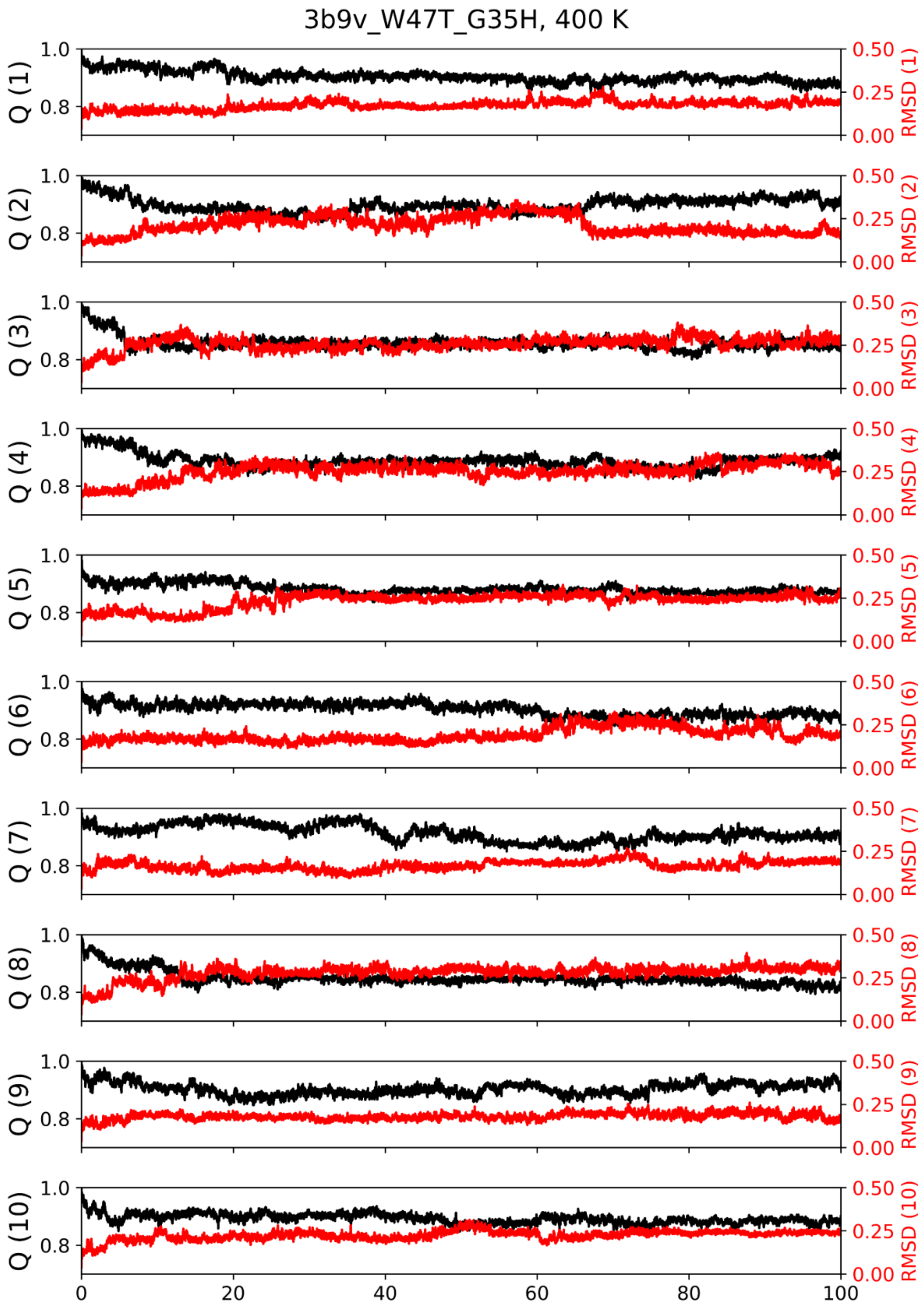

f

Figure S22: Q-value (black) and RMSD (red) versus time in ns along each parallel trajectory (Trajectory ID: 1-10) at 400 K the 3b9v W47T/G35H mutant.
